# Method matters: Comparing habitat‐ and process‐based approaches for favorability assessment

**DOI:** 10.1002/eap.70060

**Published:** 2025-06-09

**Authors:** Galen Holt, Georgia K. Dwyer, Rebecca E. Lester

**Affiliations:** ^1^ Centre for Regional and Rural Futures, Deakin University Geelong Victoria Australia

**Keywords:** ecohydrology, Murray–Darling Basin, ecological responses, habitat models, management modeling, process‐based models, wetland vegetation

## Abstract

It is common to use environmental conditions combined with habitat delineations as proxies for ecological outcomes, such as inundation of particular wetland habitats as a proxy for vegetation persistence. An alternative is to include physical environmental conditions as drivers in process‐based models that capture important events in a life cycle, thereby accounting for the environmental and biological conditions that enable those events to occur. Each approach has benefits and drawbacks and is likely to give a different assessment of the state of the target ecological responses. We modeled four iconic species of woody vegetation in the Murray–Darling Basin and considered two approaches to identifying areas favorable for each species: “habitat‐based,” the area of inundation in wetland types associated with each species, and “process‐based,” a model of the life cycle dependent on the amount, timing, and sequence of inundation and soil moisture. Calculating favorable area using inundation of identified wetland types in a habitat‐based approach provided a fundamentally different assessment to using a small number of life‐cycle processes (i.e., a process‐based approach). Further, favorable areas often did not overlap in space, with many locations found to be favorable using one method but not the other. There may be useful information to be gleaned from comparing the two, such as identifying locations of possible contraction or expansion of the species in the future. However, it is clear that the two approaches are not equivalent and care is needed in selecting an appropriate method for a given application.

## INTRODUCTION

Calls for evidence‐based decision making in natural resource management are common and widely supported (Ryder et al., 2010; Sutherland, 2006). But, while scientists possess excellent general knowledge regarding the response of ecological processes to environmental conditions, specific empirical models can be rare (e.g., with hydrology; Poff et al., 2010), particularly at a scale appropriate for management. Thus, the current state of scientific knowledge can be a barrier where data are scarce (Poff et al., 2010; Weiskopf et al., 2020), evidence has been gathered for a single location or time (Olden et al., 2014), or if it is not directly relevant for management objectives and actions (Ryder et al., 2010). Nonetheless, effective natural resource management requires an understanding of likely future conditions, where and when action might be required, and the likely ability of management actions to alter those conditions (Derepasko et al., 2023). A common solution to support decision making in water management is to use the presence of particular physical characteristics or environmental conditions (e.g., inundation, flow rates) as a proxy for ecological responses, particularly if these conditions occur within delineated habitat boundaries (e.g., wetland type, Strahler order). This approach can allow for the best available knowledge to be quickly and affordably applied, thus providing timely and evidence‐based advice to natural resource managers. Indeed, much work undertaken in fields such as ecohydrology and hydroecology are based on this approach.

The assumption that physical variables such as flow or inundation can be reliably used as a proxy is most robust where there is a mechanistic relationship between the physical variable and the ecological response (Poff et al., 2010). The flow regime is considered to be the primary driver of both aquatic and riparian ecosystems (Poff et al., 2010), where stream flow structures patterns and processes in river ecosystems (Walker et al., 1995) and flooding plays a similar role in river‐floodplain ecosystems (Van Appledorn et al., 2024). Furthermore, flow and flooding are frequently the levers by which water managers can influence water‐dependent ecosystems, so they are often a primary focus. These elements combine in many methods to develop environmental flow regimes, for example. One of the main limitations identified in setting environmental flow regimes is the available data (Tharme, 2003) and whether simple hydrologic methods can be easily applied (Leone et al., 2024). Popular methods such as the Ecological Limits of Hydrologic Alteration (ELOHA; Poff et al., 2010) have established flow–ecology relationships as the basis for environmental flow regimes (Rosenfeld, 2017). While targeted testing of flow–ecology relationships is recommended in an adaptive management approach, habitat assessments (in the terminology used here, the assessment of a physical proxy, rather than the ecological outcome itself, within identified habitat areas) are recommended where biological data are sparse and resources limited (Poff et al., 2010).

As a result, it is widespread practice for water management decisions to be based on science that utilizes habitat combined with physical proxy variables for ecological responses. For example, Mentzafou et al. (2023) reviewed the application of ELOHA in Europe and found that of the 38 assessments considered, 4 included clear assessment of biological elements, 11 had no assessment of biological elements, and 14 had no data regarding whether biological elements were assessed, instead relying on hydrology and other proxies. Indeed, it is considered an axiom of current environmental flows science that the restoration of elements of the natural flow regime will result in a corresponding restoration of the associated ecology (Arthington et al., 2018). But, available evidence suggests that the corresponding long‐term restoration of ecology can fail to follow the implementation of environmental flow regimes (and other habitat restoration approaches) (Lester et al., 2020; Palmer et al., 2010). This suggests that, in some instances at least, a focus on hydrologic and habitat‐based proxies may be insufficient, raising the question of viable alternatives and whether those alternatives lead to similar or different inference.

Habitat‐based approaches often involve identifying areas that are favorable for a species in some way (e.g., where a species has been historically located or in a wetland type known to support the species). Habitat classification methods abound (e.g., Brooks, 2021; Cowardin et al., 1979), making the identification of those “favorable” habitats straightforward in many cases. Such an approach is most likely to be effective when habitat delineation is based on well‐designed survey data and functionally relevant predictors, rather than defaulting to those variables easiest to measure (Elith & Leathwick, 2009). In some instances, these are then used as the basis for species distribution models, habitat suitability models, or even habitat selection models such as the Physical Habitat Simulation System (PHABSIM) (Elith & Leathwick, 2009; Overton et al., 2014; Railsback, 2016). As an example, species distribution models combine records of species abundance or occurrence with environmental variables to predict distributions across landscapes (Elith & Leathwick, 2009). By definition, species distribution models exclude biological processes but can involve extrapolation in space and time (Elith & Leathwick, 2009). Climate, topography, and vegetation are common environmental variables included in terrestrial models (Liu et al., 2023), while flows and inundation would be appropriate for water‐dependent ecosystems.

An alternative is to focus explicitly on ecological function and the conditions that facilitate key processes. Such models focus on the mechanisms behind a given process, providing an opportunity to capture interactions between hydrology, climatic, geological, and physicochemical conditions with ecological variables (Arthington et al., 2018). Again, there is a wide range of model types with varying degrees of complexity and data required from comprehensive dynamic population models (Gebreegziabher et al., 2023) through to simpler threshold‐based models focused on key life‐history events (Lester et al., 2020). Such threshold‐based models enable a relatively small number of ecological endpoints, corresponding to life‐history stages, for example, to be related to hydrological and other environmental thresholds (Derepasko et al., 2023). This can be an appropriate way to capture critical mechanisms without undue increase in model complexity, particularly given that data to parameterize population models are frequently absent (Arthington et al., 2018). Process‐based models are necessarily sensitive to missing important processes or mis‐specifying them as may happen with spatial extrapolation, as well as over‐inclusion of detail which can yield spurious results from slight mis‐specifications of interactions (Sutherland, 2006). Thus, process‐based approaches are most likely to be effective when able to parsimoniously capture key limiting thresholds at each life‐history stage (which may not be a trivial task; Lester et al., 2020).

Each approach has its advantages and drawbacks. In some cases, habitat‐based approaches can be as simple as identifying a set of suitable habitat types and assuming that some condition there (e.g., inundation) will equate to persistence of the species. Even in more complex applications, habitat‐based approaches are usually correlative, rather than mechanistic, with habitat fixed in place, creating significant challenges for extrapolation under non‐stationary conditions (a frequent characteristic in management‐based modeling; Arthington et al., 2018; Elith & Leathwick, 2009). Flow–ecology relationships are unlikely to remain constant with changing climates, land use, and drought and flooding cycles (Arthington et al., 2018; Rosenfeld, 2017). Moreover, management actions and outcomes are typically assessed on short timescales (often annually), so the ability to capture fluctuating conditions, rather than define a long‐run range boundary, is required (Arthington et al., 2018; Hart et al., 2021; Mentzafou et al., 2023).

Relationships between processes and environmental conditions may alter in unexpected ways outside of historical ranges, although mechanistic approaches tend to be more robust than correlative ones (Dormann et al., 2012; Sutherland, 2006). For example, important lags, covariates, and dependencies may not be captured (Thompson et al., 2018). Process‐based models often rely on large amounts of data, and it can be difficult to identify the factors limiting a given process for a given species (Lester et al., 2020). Even in simplified models, existing data are frequently limited to single (or a few) locations and a few times, making generality difficult to ensure (Olden et al., 2014). Further, observational and opportunistic data collection can lead to a weaker basis for inference than experimental manipulations (Webb et al., 2013). Careful recognition of the inherent limitations of each approach can reduce their impact on resultant inference (Dormann et al., 2012; Elith & Leathwick, 2009), but it is important to understand how each approach alters that inference (if at all).

Maintaining or improving riparian and floodplain vegetation condition, health, richness, and cover are common objectives for environmental water management, with altered flows and climate change frequently perceived as risks to sustainability (Campbell et al., 2023). Globally, water‐dependent vegetation communities have been in decline, and managers are now attempting to identify which species may be best adapted to withstand changing hydrology, and how changing species compositions may shift ecosystem function on floodplains (Tracy et al., 2024). Further, the hydrological requirements of different species can be highly variable, highlighting that the processes supporting different species can vary, even under the same inundation conditions (Tracy et al., 2024). Conversely, the extent of river water and other hydrological variables have been shown to be the primary predictors of vegetation in multiple previous studies (e.g., Berezowski & Wassen, 2023), but there are relatively few quantitative relationships developed between flooding and ecosystem dynamics compared with flow and in‐stream processes (Vaughan et al., 2009). Finally, management interventions to alter floodplain inundation characteristics have been shown to effectively improve establishment and survival of target vegetation species (Greet et al., 2022). Thus, a case can be made for taking either a habitat‐based approach or a process‐based approach to assess the likely impact of management actions, making floodplain vegetation a useful target for assessing the impact of methodological choice on the resulting inference drawn.

**FIGURE 1:**
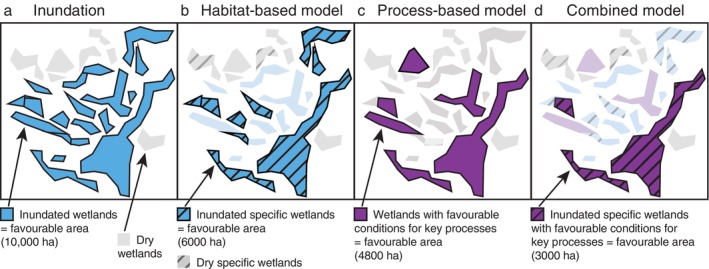
Different model types for assessing favorability for wetland vegetation. All models use wetlands as the base of the analysis, shown here as polygons, with the area of favorability determined by the area of each wetland that meets the conditions defined by the model. All models depend on inundation (a), though process‐based models have lags and also depend on soil moisture. Habitat‐based models (b) calculate the area of favorability as the inundated area of each wetland of the correct type (e.g., “red gum floodplain,” purple). Process‐based models (c) calculate the area of favorability as the area meeting inundation and soil moisture requirements for sequential life stages (here, adult survival, regeneration, and recruitment; hashed lines) within each wetland. These favorable conditions may occur in any wetland and are therefore likely to identify a different set of favorable locations compared to habitat‐based models. Moreover, life stages may be long‐lived and depend on preceding stages, so wetlands may have favorable area from process models even if they are not inundated in a given year, illustrated here in the topmost hashed wetland. Combined models (d) assess favorability by combining both habitat‐ and process‐based approaches, so the purple hashed wetlands are the spatial intersection of the habitat‐based and process‐based models. This yields the smallest favorable area; habitat‐based outcomes are reduced by requiring processes to occur within them, and process‐based outcomes are reduced by restricting them to certain habitats (e.g., wetland types). Favorable wetlands are shown in bold colors for the model of interest; dull colors illustrate areas where some, but not all, conditions are met (e.g., inundated but the wrong habitat type in b, only favorable in one model type in d). For simplicity, this figure illustrates each wetland as binary, either meeting conditions or not. In practice, the models capture the area within each wetland that meets the conditions; for example, if only half of the wetland polygon is inundated, only half of its area is counted.

The aim of this paper is to determine how the method used to link physical conditions (here, inundation and soil moisture) to species responses affects the spatiotemporal patterns of identified favorable area for a suite of floodplain vegetation species. We assess this favorability using a “habitat‐based” approach, based on inundation as a proxy for ecological outcome within defined wetland types, or a “process‐based” approach, based on simple models linking inundation and soil moisture to biological processes across life history (Figure 1). We then combine the two approaches, requiring processes to occur within habitat types, and compare the results of each. To understand the impact of the methodological choice, we compare the outcomes of the various model type (i.e., habitat‐based vs. process‐based vs. combined) for four target vegetation species. Within the habitat‐based and combined models, we consider two different definitions of favorable habitat: (1) inundation of wetland types named for the target species and (2) inundation of wetland types previously recorded as supporting the species of interest (a ground‐truthed habitat definition).

Fundamentally, habitat‐based assessments of physical proxies assume that they capture the relevant ecological outcomes. Following this logic, we hypothesize that habitat‐based assessments will yield broadly similar results to the process‐based model of vegetation recruitment due to common dependence on inundation and an expectation that habitat definitions broadly capture areas favorable for each species. That is, we expect the favorable areas to largely overlap in space and time among the methods used for each of the four species, though with some reduction in area in the process‐based approach due to specific inundation and soil moisture criteria, as well as fine‐scale differences arising from the area identified as habitat between the ground‐truthed and named habitat delineations.

The sequence of comparisons explored here enables us to identify not only how habitat‐based and process‐based models affect the assessment of favorability but also how they interact. Combined models assess how habitat requirements restrict the locations identified as favorable based on processes and how processes restrict the locations identified as favorable based on habitat type (Figure 1). Finally, we are also able to determine whether the choice of modeling approach is likely to alter the inference drawn by managers regarding where and when conditions may be favorable for a given species.

## METHODS

**FIGURE 2:**
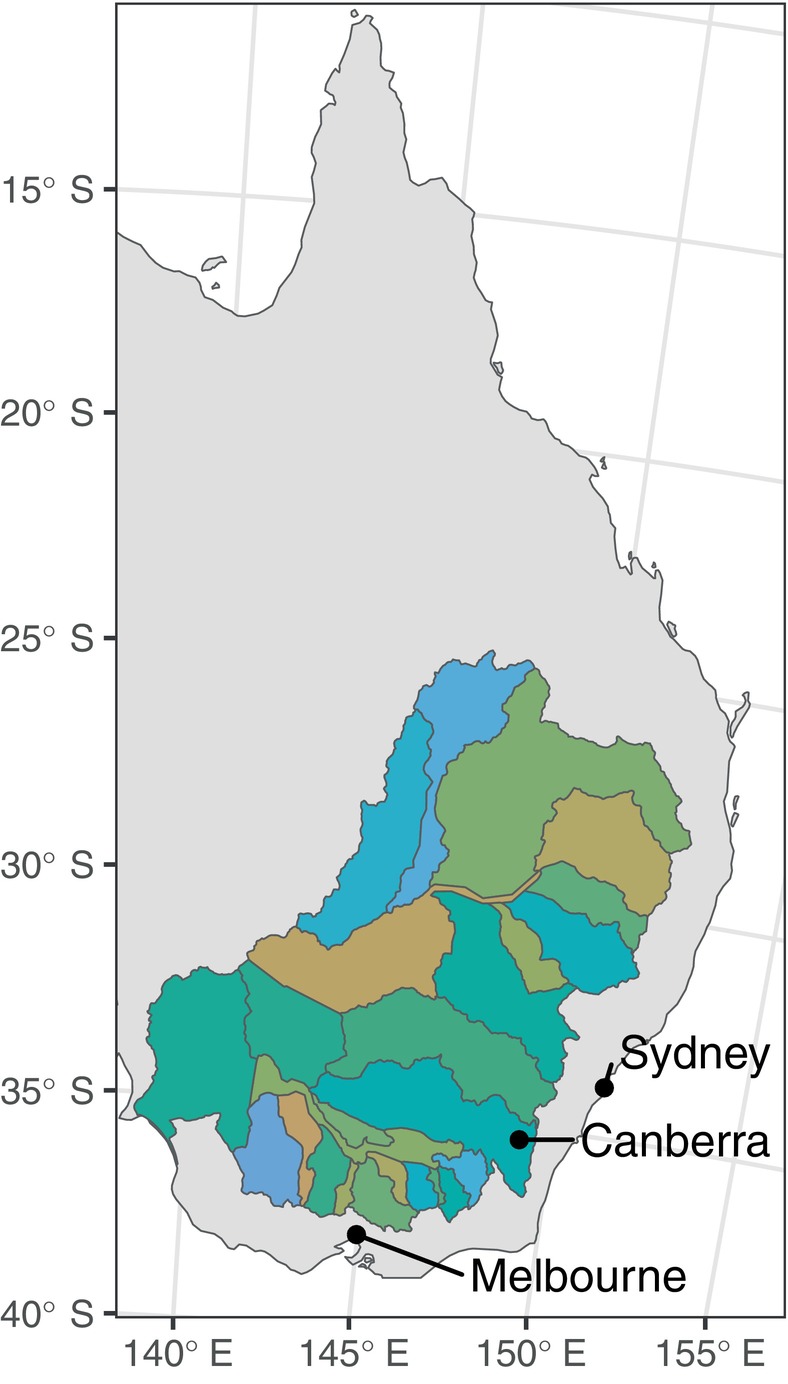
The Murray–Darling Basin in Eastern Australia, showing the 25 major catchments in the Basin, here indicated by color.

We modeled the area favorable for four iconic species of woody vegetation in the Murray–Darling Basin (Figure 2): river red gum (*Eucalyptus camaldulensis* Dehnh), black box (*Eucalyptus largiflorens* F. Muell), coolabah (*Eucalyptus coolabah* Blakely & Jacobs), and lignum (*Duma florulenta* (Meisn.) T.M. Schust.). Three of these species (river red gum, black box, and coolabah) are overstorey trees and characterize large proportions of the wetlands in the Basin, while the fourth (lignum) is a woody perennial shrub (Brooks, 2021; Rogers & Ralph, 2010). To identify the area favorable for each species of woody vegetation, we used a habitat‐based approach, a process‐based approach, and a combination of the two (Figure 1). All approaches assessed the area of favorability within the set of wetlands defined in the Australian National Aquatic Ecosystem (ANAE) classification (Brooks, 2021) in the Murray–Darling Basin, Australia (Figure 2; Appendix S1: Figure S1). This geographic dataset defines 287,680 wetland polygons, each with a designated wetland type often named for the dominant vegetation (Table 1). Using this complete set of wetlands ensures that we model only within wetlands and not uplands, thereby forming an overarching habitat filter we term “all wetlands” within which all our models are situated.

**TABLE 1:** Inundation and soil moisture requirements for four species of wetland‐dependent woody vegetation in the Murray–Darling Basin, with references.

Species	Regeneration season	Regeneration moisture duration (days)	Seedling moisture period (months)	Seedling excessive inundation (months)	Adult excessive inundation (months)	Adult inundation interval (months)	Adult inundation season
Red gum	September–December (“spring to early summer”; Johns et al., 2009; Rogers & Ralph, 2010)	14 (Casanova, 2015)	4.5 (Roberts & Marston, 2011)	>2 (“several months”; Johns et al., 2009)	24 (Rogers & Ralph, 2010)	48 (Rogers & Ralph, 2010)	July–December (“winter to early summer”; Rogers & Ralph, 2010)
Black box	May–October (northern Basin), November–March (southern Basin) (Rogers & Ralph, 2010)	10 (Jensen, 2008)	6 (Roberts & Marston, 2011)	>2 (“70 days”; Johns et al., 2009)	5 (Rogers & Ralph, 2010)	96 (8 years; Rogers & Ralph, 2010)	Year‐round (Rogers & Ralph, 2010)
Coolabah	January–April (“late summer,” “high temperature”; Roberts & Marston, 2011; Rogers & Ralph, 2010)	14 (Casanova, 2015)	6 (Inferred from other species in Roberts & Marston, 2011)	No information (Durant et al., 2020; Rogers & Ralph, 2010)	No information (Durant et al., 2020; Rogers & Ralph, 2010)	240 (20 years; Roberts & Marston, 2011; Rogers & Ralph, 2010)	No data, year‐round (Rogers & Ralph, 2010)
Lignum	September–April (“spring to autumn”; Casanova, 2015; Rogers & Ralph, 2010)	14 (Casanova, 2015)	3 (Capon et al., 2009)	>1 (any inundation during the seedling period; Capon et al., 2009; Roberts & Marston, 2011)	12 (Rogers & Ralph, 2010)	120 (10 years; Rogers & Ralph, 2010)	September–December (“spring–early summer”; Rogers & Ralph, 2010)

*Note*: Because inundation data are two‐monthly, seasonality has been approximated as closely as possible given the limitations of those two‐month units, and statements such as “several months of inundation” have been translated to >2 months. Where there was no information (e.g., excessive inundation of coolabah), we did not include those strictures. The requirements for regeneration moisture, as well as the seedling moisture requirement for lignum, were experimentally tested; all others are observational.

Our habitat‐based approach then uses a physical proxy (inundation) within a suitable subset of habitats for each species, defined in two ways: (1) the area of inundation in wetland types named for the species of interest or (2) the area of inundation in wetland types where the species of interest had been commonly recorded. Our process‐based approach used models that capture biological processes across life‐history stages to ask whether environmental conditions were favorable for those processes both individually and in sequence within each wetland, regardless of type. We then combined the habitat‐ and process‐based approaches to determine whether environmental conditions were favorable for biological processes to occur in identified wetland types and vice versa.

### Habitat‐based approach

For each species, two sets of wetland types were chosen to define potential habitat, within which inundation was a proxy for ecological favorability. First, the “named” wetland types were identified from the ANAE classification (Brooks, 2021) as those where the target species' name appeared in the wetland type description. For example, for river red gum, the matching wetland types were “River red gum forest riparian zone or floodplain,” “River red gum woodland riparian zone or floodplain,” and “Temporary river red gum swamp.” A range of similarly named wetland types were relevant to black box, coolabah, and lignum (Appendix S1: Table S1). We refer to the set of resulting polygons defining habitat for each species as the “named wetland types.”

The second set of habitat‐defining polygons, “recorded wetland types,” recognized that individuals may be found in wetland types other than those that bear the species name. To identify the set of recorded wetland types, we pulled all records from each species from the Atlas of Living Australia (ALA) (Belbin et al., 2021) using the “galah” R package version 2.0.2 (Westgate et al., 2024). The ALA provides data from a wide range of databases, with occurrence records ranging from single observations to structured multi‐year surveys and may represent presence of single or multiple individuals at a location (Belbin et al., 2021). We intersected those records with all ANAE wetland polygons and identified all wetland types where each species occurred. We used this set of types with documented occurrence to define suitable habitat, retaining all polygons of each identified type not just the individual polygons which included an ALA record due to the uneven effort in sampling across the Basin to generate the ALA records. Hence, the set of recorded wetland types for river red gum includes the three named wetland types mentioned above in addition to “Black box woodland riparian zone or floodplain” and “Permanent lake,” for example, along with 14 others (Appendix S1: Table S1). To avoid selecting spurious matches for recorded wetland types, we selected only those wetland types with >0.5% of the records for that species. For black box and coolabah, this led to exclusion of one named wetland type in each of the recorded wetland types (Appendix S1: Table S1).

Thus, for each species, we consider two habitat‐based models, one for each set of wetland types: “named” (defined by the species' name in the wetland type description) and “recorded” (defined by wetland type with >0.5% of ALA records for that species). The supplementary material includes the counts of ALA records in each of the ANAE wetland types (Appendix S1: Table S1, Figures S1–S3), as well as their spatial distribution across the Murray–Darling Basin (Appendix S1: Figure S4).

### Process‐based approach

Life‐cycle process‐based models were developed with a strictures‐and‐promoters framework (Lester et al., 2020), which is a simplified process‐based model especially suitable for situations with limited knowledge. This approach captures the major known limitations (“strictures”) and requirements (“promoters”) of species while also maintaining a life‐cycle focus. The focus on life cycles gives the opportunity to identify issues such as the most‐limiting life stage and understand why apparently favorable conditions at one life stage may not lead to positive outcomes for populations. These models were based on inundation and soil moisture within all individual wetlands defined in the ANAE.

Three primary life stages for each species were modeled: adults, regeneration (germination or sprouting), and seedling survival. For each species, we identified several major environmental requirements for each life stage from the literature (Table 1). The state of the available science means that the majority of these requirements are based on observational rather than experimental evidence. The length of time moisture is required for germination for all species and for lignum seedling survival are the only experimentally‐derived requirements; all others are observational (Capon et al., 2009; Casanova, 2015; Jensen, 2008). Where no information was available for an individual species for a given life stage, no threshold was applied. For example, due to a lack of information, coolabah seedlings had no inundation stricture and are modeled as surviving any length of inundation during their seedling period (Table 1). This almost certainly overestimates coolabah seedling survival. Favourability for adult condition and survival depended on inundation, with strictures arising from excessive inundation (continuous inundation can waterlog or drown roots), the interval between inundation events (most species require inundation at some frequency), and the season in which those events occur. For regeneration, all species required preceding inundation followed by sustained duration of optimal moisture over several days, sometimes in a specific season. The seedling stage then extended this requirement for soil moisture over months to allow growth past the cotyledon stage and establishment of robust root systems. While each of these species needed recurrent flooding for establishment and persistence, the seedling stages tended to be sensitive to excessive flooding; hence, we also included seedling failure with prolonged inundation.

Conditions favorable for each life stage were assessed in the sequence of the life cycle within each ANAE wetland. For example, conditions may be favorable for seedling survival, but if there was no preceding germination, then that area would not be identified as favorable for recruitment. For the species included in this study, adults can be very long‐lived (i.e., up to hundreds of years) and do not have persistent soil seed banks (Roberts & Marston, 2011). Thus, we considered the “reference” stage of the population cycle to be adults. To incorporate the life cycle into the stricture calculations, regeneration (germination or sprouting) required the presence of adults to provide seeds or stems, and recruitment required successful regeneration to provide the initial stock of growing seedlings. Conceptually, new adult recruitment would require survival of these seedlings. However, due to the relatively short time series of available data compared to adult persistence and the simplicity of the model, we did not create a fully dynamic population model. Instead, we interpreted the outcome of the adults–regeneration–recruitment sequence as favorable conditions for recruitment to the persistent adult stage. For clarity, all results presented here illustrate the stages in sequence.

### Environmental data

We used two publicly available datasets to establish environmental conditions: a two‐monthly inundation depth layer (30 m pixels; Teng et al., 2023) and a daily soil moisture layer (0.05° pixels; Frost et al., 2018), chosen to capture the environmental requirements of the species, with inundation also being a common hydrological proxy of favorability. Both of these datasets are raster time series and were processed into the set of 287,680 wetland polygons comprising the wetland types (Brooks, 2021) spanning the entire Murray–Darling Basin. Processing rasters into polygons yields several advantages stemming from the wetland type classifications. The wetland polygons are defined by wetland type and so account for similar conditions over areas of different sizes and shapes, in contrast to gridded raster data. Moreover, areas outside of wetlands can be ignored. This allowed much faster processing (e.g., compared with standard grid methods) while dynamically adjusting grain size to match ecologically relevant habitat units (see Holt et al., 2024). We processed the rasters using the eFlowEval R package (Holt et al., 2024), yielding inundation and soil moisture time series in each polygon. For inundation, the area inundated to a depth >5 mm was calculated for each polygon. For soil moisture, the area of each polygon with soil moisture between 10% and 30% was calculated, reflecting soil moisture requirements for recruitment and seedling survival (Jensen, 2008).

The two‐monthly timestep of the inundation layer created challenges, given that the excessive inundation thresholds for seedlings were less than or similar to that interval, while some other thresholds, for example, black box adult inundation, could not be evenly divided into two months. Finer‐resolution data would enable a greater degree of certainty regarding likely seedling survival but were not available. However, given the focus of this work was to compare underlying approaches, our conclusions regarding the similarity or otherwise of the resulting areas are highly unlikely to be affected by this assumption given that those assumptions are consistent among the approaches compared.

### Assessing favorability

All assessments were undertaken using the eFlowEval R package (Holt et al., 2024), which provides functions and workflows for specifying models of favorability that might depend on sequential life stages. Specific parameterization of both the habitat‐based and process‐based models for these species was developed (code available at Holt & Dwyer, 2025), yielding modeled areas of favorability for each species and model type. This package was further used to aggregate the results to yearly timescales and to both catchment and basin scales for presentation and analysis. All analyses were done for each species independently, capturing their different habitats and life‐stage requirements.

To assess habitat‐based favorability, we used inundation as a proxy for ecological favorability, provided it occurred in appropriate habitat types. We started with the areas of inundation in every wetland polygon obtained from processing the inundation data and set those areas to zero unless the polygon represented one of the named or recorded wetland types. Thus, we were left with two outcomes for each species: the area of inundation in named wetland types (with the species' name in the wetland type description) and the area of inundation in recorded wetland types (defined by wetland type with >0.5% of ALA records for that species). These form the habitat‐based assessment.

To assess process‐based favorability, we constructed a stricture‐based model that assessed favorability for adults, germination, and seedling survival, using the parameter values for each species shown in Table 1. These conditions were assessed at each timestep in each wetland polygon for each species. To do this, we calculated the area of favorable conditions in each wetland polygon at each timestep available in the environmental data (daily for soil moisture, two monthly for inundation), as determined by the relevant models.

The requirements necessarily included not just the current conditions on each day or two‐monthly period, but also preceding conditions (e.g., soil moisture needed to be maintained for some specified number of days). Thus, in this example, the favorability of each day was determined by whether the preceding required number of days had met the soil moisture requirement. Further, each life stage was conditional on the preceding stages. So, for conditional germination to be successful, the germination conditions must be favorable, but there also must have been favorable conditions for adults preceding that germination (typically 10–14 days, Table 1), as these species tend to regenerate from an aerial seedbank or sprouting (Roberts & Marston, 2011). Likewise, successful recruitment requires not only conditions favorable for seedling survival (an extended period of moist soil during which time there is no excessive inundation) but also conditions favorable for adult survival and germination at the start of the seedling period so that there are seedlings to survive. In these cases, the eFlowEval framework looks back to the start of the life stage for the favorable area in the preceding stage. The favorable area for a stage conditional on preceding stages is thus the minimum of the preceding stage area at the start of the target stage and the favorable area of the target stage. From these analyses, we return the area of favorability from regeneration following adults, as well as for recruitment as the full sequence from adults to regeneration to seedling survival. These form the process‐based results.

Where identified thresholds were less than a single two‐monthly inundation timestep, we assume that inundation within the two months was continuous, which overestimates true inundation. This is an optimistic assumption in the case of many regeneration requirements, where soil moisture must be met for some small number of days following inundation, but a conservative assumption for lignum seedling survival, where inundation >1 month yields mortality (Table 1). Similarly, where identified inundation thresholds did not divide evenly into two‐month periods, we rounded up to the two‐month period. This assumption was conservative or permissive depending on the stricture in question. For example, a five‐month excessive adult inundation for black box required three inundation periods to cause mortality, thereby overestimating survival. By contrast, red gum seedlings require 4.5 months of soil moisture, during which time there cannot be inundation greater than 2 months. Rounding up from 4.5 to 6 months of no excessive inundation gives a longer period over which inundation might occur, thereby overestimating mortality (Table 1).

To assess the combined impacts of habitat‐based and process‐based requirements, we took the life‐stage process results in each wetland polygon and set the area of favorability to zero unless the polygon was a named or recorded wetland type for each species, that is, the spatial intersection of the habitat‐based and process‐based models. This step was repeated twice for each species, once for each of the named and recorded sets of wetland types. The result was an area of favorability meeting both habitat‐based and process‐based requirements. If process‐based requirements were not met in a given wetland, the favorable area would be zero, and if habitat‐based requirements were not met, the favorable area would again be zero.

Critically, these combined results are the area within the wetland types representing known habitat, where process‐based requirements were met because we used the favorable area based on the life‐stage process requirements, not just the area of inundation. These results from the combined model provided the basis for our primary analyses, providing a point of comparison to each of the areas identified as favorable using a habitat‐based or a process‐based approach separately.

All outputs produced time series of the area of favorability in each of the 287,680 wetland polygons. While this allows granular modeling as close as possible to the scale of biological processes, for interpretation these areas were then averaged over the water year (July 1–June 30) and summed over space (either catchment or the whole Basin). Given the nature of the inundation data, averaging over the water year bears caution. The inundation data are the maximum inundation in each two‐monthly interval; thus, we do not know how long the water was at that level within the two months, and the average of six maxima is an unusual statistic. However, this was chosen as it gives the best holistic picture available of the extent of inundation in that year. Ongoing inundation (>2 months) is better captured by this metric than by the maximum of the maxima, which would be another reasonable choice. Ultimately, these choices were only made for visualization; all modeling was done at the scale of the input data, and using another statistic, such as the yearly maximum, did not alter the broad findings based on our sensitivity testing (results not shown). The sums over the catchment or Basin simply added up the favorable area in each wetland within the larger spatial unit, giving the total favorable area in the catchment or Basin in hectares.

To aid visualization, the axes or colors representing area (in hectares) are presented on the log scale in all figures. This is because of the large range in values in mean area produced by the different comparisons, which are very difficult to see in their native units. Thus, the log scale enables better visual comparison between very large numbers (e.g., for adult persistence) compared with very small numbers (e.g., for recruitment) which are otherwise difficult to represent on the same axis. All analyses were undertaken in the native units, and results presented in the main text and tables are given in hectares.

### Analyses

Our analyses explore the favorability for each species as determined by each type of model (habitat‐based, process‐based, and combined). Habitat‐based and combined models included each of the named and recorded wetland type specifications. Process‐based and combined models included the different stages in the life cycle. Our primary interest here was in comparing the outcomes of the different modeling choices. We performed these comparisons at different scales (for each of 25 catchments and for the whole Murray–Darling Basin [Appendix S1: Figure S5], yearly and over the full available period of data 1988–2022).

All outcomes are based on historical time series, so each model type yields a single time series of modeled favorability data. This means that there are no replicates appropriate for statistical testing, and results are presented as direct comparisons. Where variance is shown, for example, in boxplots, we do so to illustrate the distribution of data and the (often substantial) variation in outcome in different years, not the distribution of a set of independent samples. The statistical analysis of modeled results is rarely appropriate without the opportunity to undertake stochastic runs due to a lack of independence. However, the scale of the differences identified between approaches is large, so direct comparison is informative, particularly since all models use the same inundation time series.

The measurements of favorable area from the process‐based models provide a measure of realized environmental tolerance; species or life stages with wider environmental tolerances according to the process definitions in Table 1 will have greater favorable areas. To assess whether the impact of model choice differs depending on the environmental tolerance, we assess the fraction of the favorable area according to process models that is also favorable according to habitat models. The combined model outputs (i.e., the favorable area for processes that occur within habitat types) capture the area of overlap between the model types, but this would be expected to scale with the overall favorable area. Since the key question is whether a greater proportion of the process‐based favorable area is shared between model types with increasing environmental tolerance, that is, a disproportionate increase in overlap, we divide this overlapping area by the area from the process model alone. We then assess whether this proportion increases with the size of that favorable area (tolerance) as tolerance varies across species and life stages. Increases in this proportion would indicate a greater concordance between model types.

## RESULTS

**FIGURE 3:**
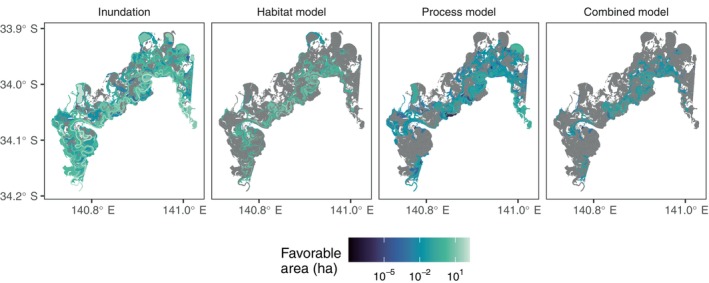
Illustration of favorability outcomes (in hectares) for all 1167 Australian National Aquatic Ecosystem (ANAE) polygons in the Riverland wetland complex Ramsar site for the water year beginning July 2013. Zero favorability is shown in gray for clarity. Inundation is simply the area of inundation. The habitat‐based model then limits this to the “named” wetlands, so while those wetlands' favorability equals their inundated area, many wetlands are not of the specified type. The process‐based model requires favorable conditions for regeneration, with the resulting favorable area lower than the inundated area because of the timing and soil moisture requirements. In the combined model, which requires favorable conditions for regeneration within named habitat types, the set of ANAE wetlands with any favorability partially overlaps between the habitat‐based and process‐based models, yielding the smallest set of wetlands with any favorability. Where wetlands are favorable, their area of favorability equals that of the process‐based model.

Each ANAE wetland in each year yielded an area of favorability for each model. We illustrate this outcome for red gum in the Riverland wetland complex Ramsar site in the Lower Murray catchment in South Australia, which contains 1167 ANAE polygons (Figure 3; see Appendix S1: Figure S6 for location in the Basin). In this illustration of the year starting July 2013, inundation is widespread, though there are some wetlands that are not inundated. Those wetlands that are inundated have a range of inundated area. The habitat‐based model limits consideration to only wetlands with “red gum” in their name; hence, while those named wetlands have a favorable area equal to their area of inundation, many inundated wetlands are no longer favorable. The process‐based model (here, regeneration) shows favorability for regeneration occurring in a range of wetlands, though the area of favorability tends to be less than the area of inundation due to the timing and soil moisture requirements of regeneration (Table 1). Moreover, the set of favorable wetlands shows partial overlap with those in the habitat‐based model; some wetlands have favorable area in both models (e.g., in the central areas), while others are favorable in only the process‐based models (e.g., areas away from the river in the top right), and some are only favorable in habitat‐based (e.g., bottom left). The overlapping areas are the outcome of the combined model, which is the area favorable for processes within the habitat restrictions. Such outcomes are found for each ANAE wetland across the Basin in each year and then summed spatially or averaged through time to conduct all following analyses.

**FIGURE 4:**
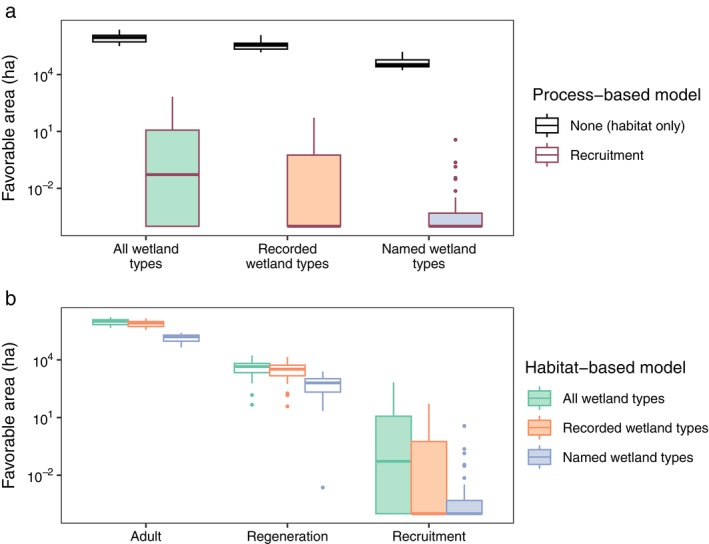
Favorable area (in hectares) for river red gum for all models at the basin scale. Panel (a) shows the differences between habitat‐based models (open boxes, a), as well as the combined models of recruitment within each habitat type (red outlines, a). Panel (b) shows differences between process‐based models at each successive life stage dependent on inundation, soil moisture, and their timing (green boxes, b; Table 1) and combined models requiring those processes to occur within named (blue) or recorded (orange) wetland types. All models are restricted to all Australian National Aquatic Ecosystem (ANAE) wetlands, “all wetland types.” Filled boxes are identical between panels to aid comparison. Boxes show the distribution of values at the basin scale over time, with the underlying data being the basin‐scale time series of favorable hectares. Central bars are the median, upper and lower box boundaries are the first and third quartiles, whiskers go to the furthest data point within 1.5× the interquartile range (IQR), and dots are data values beyond 1.5× IQR. Thus, the boxes show the distributions resulting from temporal variation, not independent replicates, and are not used for statistical inference. The *y*‐axis is on the log scale to improve visualization of large differences in favorable areas. For mean values of the data underlying each box, see Table 2.

For river red gum, using the overarching filter of all wetlands, the area identified as favorable using either inundation as a physical proxy or the process‐based adult survival was similar on average over time (964,994 vs. 1,034,448 ha, respectively; Figure 4a,b, Table 2). Adult survival can have larger areas of favorability because large inundation events can maintain favorability for several years (Table 1). Using the two habitat‐based models incorporating wetland type constraints (i.e., either using named wetland types [defined by the species' name] or recorded wetland types [defined by wetland type with >0.5% of ALA records for that species]) reduced the area favorable compared with all inundated wetlands (by 95% or 60% respectively, noting the log scale; Figure 4a, Table 2). Further imposing process‐based constraints (i.e., the combined models requiring recruitment favorability within each set of wetland types) then dramatically reduced the favorable area (by >99% compared to each habitat‐based model alone; Figure 4a, Table 2). This reduction means that many areas that were inundated and of appropriate wetland type (and so met habitat‐based requirements) did not also meet requirements for life‐cycle processes.

**TABLE 2:** Mean area (in hectares) over time in the Basin of red gum favorability in habitat‐based and process‐based models.

	Habitat inundation	Adult	Regeneration	Recruitment
All wetland types	964,993.70	1,034,447.59	4882.39	46.09
Recorded wetland types	382,198.50	850,262.60	3913.26	2.73
Named wetland types	45,161.23	143,202.56	775.46	0.14

*Note*: “All wetland types” is the overall set of all wetlands in the Australian National Aquatic Ecosystem (ANAE) and forms the set of locations used for all models. Habitat‐based models are defined by various wetland restrictions, with “Recorded wetland types” being the set of wetlands of type recorded to have species presence in the Atlas of Living Australia [ALA] and “Named wetland types” being the set of wetlands with types named for the species. The “Habitat inundation” column is the case where inundation is used as a proxy for favorability within each set of wetlands, giving the habitat‐based model results. The remaining three columns are the three stages of a process‐based model, with the top row giving the outcomes for “process‐based” models and the second and third rows giving outcomes for combined models including the habitat restrictions. All values are mean hectares over time and so are the mean value of the data underlying each box in Figure 4. Note that the process‐based areas for adults can be larger than habitat inundation areas within a given set of wetlands because they depend on infrequent, large, inundation events that maintain high adult favorability through several subsequent years.

When considering process‐based models alone (Figure 4b), the area favorable for adult survival was greatest (1,034,448 ha; Table 2). Substantial area within that was also favorable for regeneration (germination or sprouting conditional on preceding adult favorability; 4882 ha on average over time; Figure 4b, Table 2). The amount of favorable area was considerably lower when conditions required for seedling survival (and thus “recruitment”) were included, with recruitment therefore being the limiting life stage (46 ha on average over time; Figure 4b, Table 2). For each sequential step in the life cycle, adding wetland type limitations (i.e., the combined habitat‐ and process‐based models; Figure 4b) further reduced the area favorable in each case (e.g., by 18% and 86% for adult survival in recorded and named wetland types, but 94% and >99% for recruitment; Figure 4b). Thus, many areas identified as favorable based on life‐cycle processes fell outside named wetland types or recorded wetland types.

These trends were also apparent for the other species investigated (Appendix S1: Figures S6–S8, Tables S4–S6). In all cases, more area was identified as favorable in habitat‐based models considering inundation within wetland type classifications (named wetland types and recorded wetland types) than in process‐based models considering regeneration and recruitment processes. In all species, seedling survival was the limiting factor, usually dramatically reducing the area favorable for a species compared with any other life‐cycle stage or wetland type classification (by >99% for all species; Table 2; Appendix S1: Tables S2–S6). All species showed loss of favorable area from process‐based to combined models (where process‐based models were constrained by wetland type), though this varied dramatically depending on the species, stage, and constraint (e.g., lignum recruitment favorability declines by >99% when restricted to named types, but only 55% when restricted to recorded types, while coolabah adult favorability declines by 73% and 54%, respectively). Black box and coolabah had very little area favorable for recruitment relative to the other species (9.6 and 0.9 ha, respectively; Appendix S1: Figures S6 and S7, Tables S4 and S5). For coolabah, the area favorable for recruitment was little more than zero across the available record. Lignum had the largest area favorable for recruitment, but little of that was in named wetland types (<0.1 ha of a total of 143 ha; Appendix S1: Figure S8, Table S6). By contrast, lignum recruitment showed much greater overlap with recorded wetland types (55% of all areas favorable for lignum recruitment occurred in recorded wetland types) due to its presence in many wetland types which do not bear its name (Appendix S1: Table S1).

**FIGURE 5:**
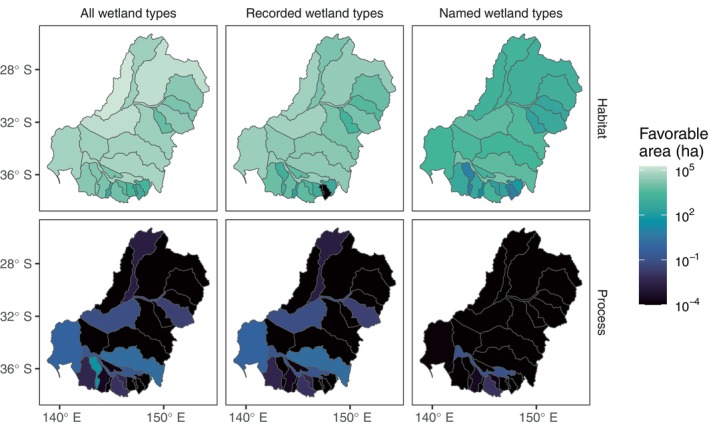
Spatial variation in the area (in hectares) favorable for river red gum based on inundation within habitat delineation (top) or according to process‐based and combined models (bottom). Habitat‐based models rely only on the area of inundation within all wetland types (top left), recorded wetland types (top middle; defined by wetland type with >0.5% of Atlas of Living Australia [ALA] records for that species), and named wetland types (top right; defined by the species' name). Process‐based models require conditions favorable for recruitment, constrained to all wetlands (bottom left), recorded wetland types (bottom middle), and named wetland types (bottom right). Life‐cycle processes include adult survival, germination, and seedling survival in sequence (referred to as “recruitment”). Favorable area represents an average through time for each catchment.

There was substantial variation in how the reductions in favorable area for red gum manifest in space (Figure 5; Appendix S1: Table S7). The greatest area of inundated wetland tended to occur in the larger catchments in the central and northern parts of the Basin (e.g., Condamine–Balonne and Barwon–Darling; catchment names in Appendix S1: Figure S5). The habitat‐based model considering only inundation in recorded wetland types resulted in a relatively small decline in favorable area compared to all wetlands (as it included many wetland types and thus more area overall), while inundation only in named wetland types restricted the favorable area across the whole Basin (a 95% decrease in total over the whole Basin; Figure 5; Appendix S1: Table S3). Catchments were disproportionately affected, ranging from nearly 100% loss in Avoca to 84% loss in the Goulburn. Four catchments (Condamine–Balonne, Mitta Mitta, Paroo, and Avoca; catchments in both the northern and southern Basin) had losses greater than 99%, and four had losses less than 90% (Goulburn, Central Murray, Edward Wakool, and Kiewa; all in the southern Basin) (Appendix S1: Table S8, catchment locations in Appendix S1: Figure S5).

Much of the area identified as favorable for red gum in process‐based models (Figure 5 bottom left panel; Appendix S1: Table S7) occurred in the southern Basin but represented a large loss in favorable area compared to inundation in all wetland types (Figure 5 top left panel), noting that processes across the full life cycle were included for this analysis. Only three catchments (Lower Murray, Murrumbidgee, and Avoca) had more than 0.001% as much favorable area for recruitment as inundation in all wetlands, with Avoca having the most at 0.2% (Figure 5; Appendix S1: Table S7). The combined model considering recruitment within recorded wetland types gave a relatively similar pattern of favorable areas to the processes alone, while the combination of recruitment and named wetland types dramatically restricted the favorable area to a small number of catchments in the southern Basin, with the Central Murray having the vast majority of area at only 0.13 ha (Appendix S1: Table S7).

Again, these trends were broadly consistent across the other three species investigated (Appendix S1: Figures S9–S11). In each case, there were areas of the Basin that were disproportionately affected using each method for determining favorable area. As noted above, coolabah had nearly no area favorable for recruitment and all recruitment favorability (8813 ha) occurred in the Avoca catchment. The Avoca is outside the range of the species, which is typically restricted to the northern Basin (Appendix S1: Figure S2), and has no recorded or named wetland types for coolabah (Appendix S1: Figure S10). Interestingly, while there are named coolabah types in the Murrumbidgee that had some recruitment (“Temporary coolabah swamp”), this type had only a single record in the ALA (Appendix S1: Table S1; and only a single coolabah record was seen in the catchment, Appendix S1: Figure S3) and so only using recorded types restricted this species to its typical range in the northern Basin (Appendix S1: Figure S10). Other species also had ranges that did not span the whole Basin but were more widespread (Appendix S1: Figures S9 and S11), so the impact was less dramatic.

**FIGURE 6:**
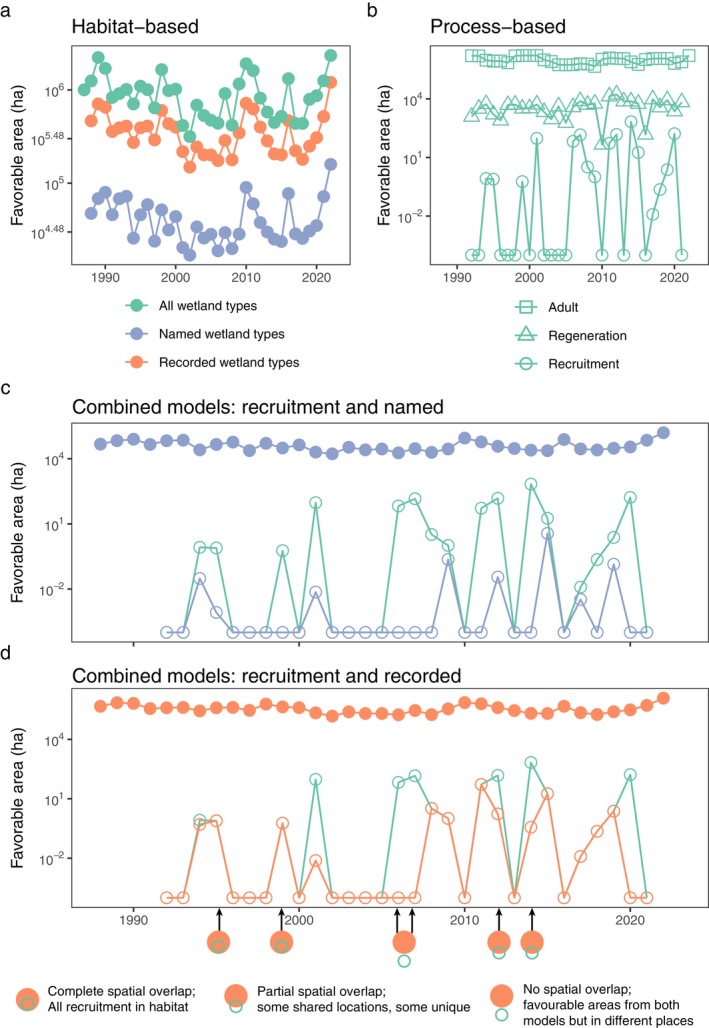
Temporal variation in the area (in hectares) favorable for red gum based on (a) habitat‐based models assessing inundation of all wetland types, named wetland types (defined by the species' name) and recorded wetland types (defined by wetland type with >0.5% of Atlas of Living Australia [ALA] records for that species), (b) process‐based models assessing favorable conditions for life‐cycle processes dependent on inundation and soil moisture in all wetlands, and combined models, including comparisons with the habitat‐ and process‐based models (c, d). Three sequential life stages are shown in (b): adult survival; adult survival and germination (referred to as “regeneration”); and adult survival, germination, and seedling survival (referred to as “recruitment”). Recruitment is the final stage of the life cycle, so it is used to assess the outcome of process‐based models in most other figures and text. The time series for process models begin later than the beginning of the inundation record due to dependence of adult favorability on inundation over several preceding years and so cannot be assessed near the beginning of the time series. Panels (c) and (d) compare the favorable area from habitat‐based (filled circles) and process‐based (green open circles) models alone, as well as their combination, (c) named wetland types (open blue circles) and (d) recorded wetland types (open orange circles). The combined models in (c) and (d) (blue and orange open circles) require both habitat‐based and process‐based recruitment conditions to be met, that is, recruitment occurring within the named or recorded habitat types. Thus, they represent the area of spatial overlap of the two types of models. Different overlap situations are conceptualized below the plot, with examples indicated in panel (d). Years when the open green circles exactly match the blue or orange open circles show completely nested spatial overlap—all wetlands favorable for recruitment lie within the named or recorded types. When the blue or orange open circles are at the bottom of the axis but the green open circles are not, then there is no overlap; both habitat‐ and process‐based models find favorable locations, but they are not in the same wetlands. The intermediate case, where the blue or orange open circles are lower than the green open circles, shows partial spatial overlap—some wetlands are favorable for both processes and are a selected habitat type, while other wetlands are the right type but not favorable for processes, and others are favorable for processes but not the right type.

The patterns of favorability through time show broadly similar outcomes in terms of areas of favorability among the models, with habitat‐based models always having several orders of magnitude more favorability than process‐based models. However, their comparison identifies important features of their consistency and interaction in combination (Figure 6; Appendix S1: Table S9). The area identified as favorable in habitat‐based models tended to follow similar temporal trajectories in each of the sets of wetland types (Figure 6a). Because these models depend only on inundation, they tend to move together as total inundation varies between years, with overall differences largely caused by the area of wetlands in the different categories (e.g., named wetland types are a smaller set of wetlands, so consistently have lower favorable area than all or recorded wetland types, but in years with high inundation [i.e., 2010], all habitat categories have increased favorability, while in years with low inundation [i.e., 2015], favorability is relatively low for all; Figure 6a). By contrast, the area favorable for each life stage was broadly independent of each other stage, and each stage had noticeably different variation across years, so the area favorable for all stages combined was more variable temporally (Figure 6b). The two initial stages, adults and regeneration, tended to have far higher and more constant favorable area than recruitment, indicating that seedling survival was limiting and most sensitive to fluctuations in environmental conditions.

Of particular interest was the finding that, through the time series, wetland types or life‐cycle processes imposed constraints in different years. Habitat‐based models always yielded larger favorable areas than process‐based recruitment models. However, the favorable area when both habitat‐ and process‐based models were considered together was variable and determined by the degree of spatial overlap between model types. These overlaps exhibited notable variation among years, and so the spatial congruence of the habitat‐ and process‐based models differed temporally as well as between the named wetland types and the recorded wetland types (Figure 6c,d, respectively). For example, in 1995, the area identified as favorable based on life‐cycle processes was fully contained within recorded wetland types (0.8 ha; Figure 6d; Appendix S1: Table S9), indicating that processes were the primary factor limiting favorable area. A similar pattern arose in 1999. In other years, habitat and process requirements jointly limited the area of the combined model. For example, in 2012, there was incomplete overlap in the area identified as favorable using either wetland types or life‐cycle processes (1.7 ha was jointly favorable, within a total of 151 ha favorable for recruitment and 399,998 ha of inundated recorded types; Appendix S1: Table S9), suggesting that each was limiting in some locations. In 2006 and 2007, there was high favorability in both recruitment (67 and 145 ha) and recorded habitat (174,601 and 286,358 ha) when considered separately but no favorable area when considered together (Figure 6d; Appendix S1: Table S9). Thus, in these years, there was no spatial overlap between the two model types; all inundation of recorded types occurred in areas unfavorable for recruitment and vice versa. Using named wetland types yielded lower overlap than recorded, for example, complete overlap with recorded types in 1995 was only partial with named types, and in 1999, recorded types had complete overlap, while there was no overlap with named wetland types (Figure 6c,d).

The other species showed broadly similar patterns, in that each had more favorable area identified in recorded wetland types than named wetland types (Appendix S1: Figures S12–S14). For each, seedling survival was the limiting step, and in all cases (except coolabah), the combined models had years with recruitment completely nested within wetland type, as well as years with partial and no spatial overlap (Appendix S1: Tables S12–S14). For example, black box showed recruitment contained within recorded habitat types in 2002 and 2008, lignum recruitment and recorded habitats had partial overlap from 2006 to 2008, and favorable wetlands were entirely separate between both habitat‐based models and recruitment for coolabah in 2014. There were also some interesting nuances across species. For black box, there was no overlap between areas found to be favorable based on named wetland types and those based on life‐cycle processes despite six years with some recruitment favorability (Appendix S1: Figure S12c). This was not the case for recorded wetland types, where the area of recruitment favorability was completely contained within the recorded types in all but two years (Appendix S1: Figure S12d). Conditions were favorable for coolabah recruitment only once in the available time series, and the area identified did not overlap with either named or recorded wetland types (Appendix S1: Figure S13). Conditions were frequently favorable for lignum recruitment in both habitat‐ and process‐based models (Appendix S1: Figure S14). However, the wetlands identified as favorable for recruitment only overlapped with named wetland types in 2007, 2014 (very little), and 2019 but were little constrained by (had high overlap with) recorded wetland types (Appendix S1: Figure S14c,d).

Coolabah and lignum have the lowest and highest recruitment success, respectively, because of their soil moisture requirements, but contrary to inundation requirements. Lignum has the most restrictive inundation requirements (seedling mortality occurs with any inundation), while coolabah is modeled as having no inundation stricture for seedling survival (no amount of inundation causes mortality; Table 1). However, their seedling survival rates run counter to these inundation limitations because lignum requires only three months of soil moisture following germination, while coolabah requires six months.

**FIGURE 7:**
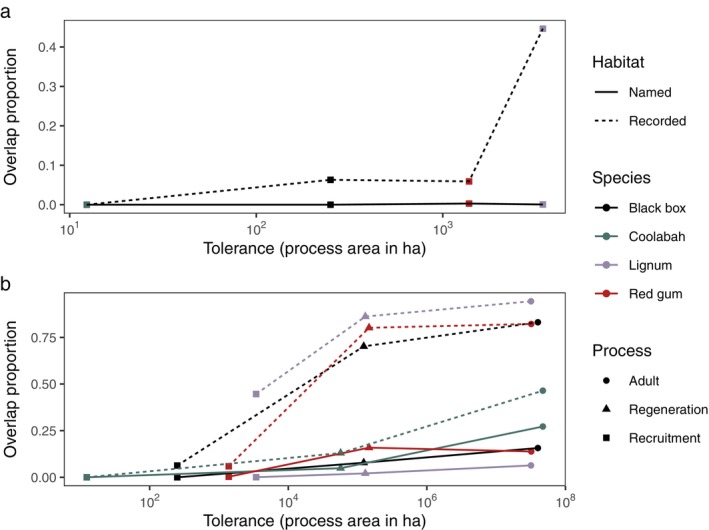
The proportion of favorable area for recruitment within appropriate named (solid) and recorded (dashed) wetland types, that is, the combined models, compared with environmental tolerance assessed as the favorable area for life‐cycle processes. Tolerance varies across species and life stages due to their different dependence on inundation and soil moisture in Table 1. Panel (a) focuses on the between‐species differences in recruitment, while panel (b) focuses on the within‐species but across‐stage differences. Overlap proportion captures disproportionate changes in the overlap between habitat‐based and process‐based models, with larger values meaning more congruence between the model types as more of the areas favorable for processes fall within defined wetland types (named, solid; recorded, dashed).

The four species differed dramatically in environmental tolerances, as measured by favorable area from process‐based models taken to the final life stage of recruitment, ranging from 0.9 ha for coolabah to 143 ha for lignum (Figure 7; Appendix S1: Tables S2 and S3). This area of favorability was 2–6 orders of magnitude smaller than in the habitat‐based models (Figure 4; Appendix S1: Figures S12–S14, Tables S2 and S3), and so for all species, the choice of model had dramatic impacts on the outcome. Comparing the spatial overlap of process‐based and combined models shows a trend of increasing concordance between the locations favorable for processes and recorded wetland types as species' environmental tolerances increase (from 0% of area favorable for coolabah recruitment falling in recorded wetland types to 45% for lignum; Figure 7a). However, this pattern is not seen for combined models using named wetland types, where the overlap remains a consistently low proportion of the favorable area for recruitment for all species (maximum 0.3% for red gum). These patterns are similar when considering environmental tolerances across life stages within each species. Life stages with high environmental tolerance (adults and regeneration) have a much greater proportion of their favorable area within recorded habitat types than does recruitment (e.g., 6% of the area favorable for black box recruitment falls in recorded wetland types, while 83% of area favorable for black box adults does; Figure 7b). For all species, this relationship is weaker when the overlap with named habitat types is considered (e.g., black box overlap with named habitats is only 16% of the favorable area for adults).

## DISCUSSION

When assessing the relative condition of species of interest in a broad spatial area, it is common to assume that meeting key environmental requirements (e.g., inundation) in identified suitable habitat (e.g., recorded wetland types) is sufficient to demonstrate areas favorable for that species. This is despite many methods and reviews specifying the need to simultaneously consider ecological processes (Arthington et al., 2018; Rosenfeld, 2017). Here, we directly compared the area identified as favorable for a habitat‐based approach and a process‐based approach in turn and then combinations of the two for four vegetation species in the Murray–Darling Basin, Australia. We discovered that calculating favorable area using inundation of identified habitat provided a fundamentally different assessment from that generated using a small number of life‐cycle processes. Further, the areas identified as favorable by each method often did not overlap, with many locations found to be favorable using one method but not the other. As a result, combining habitat‐ and process‐based methods provided the smallest estimates of favorable area. Thus, the outcome of the assessment of favorable area for a particular species is highly sensitive to the method selected.

While hydrologic variables such as flow and inundation have been identified as being key drivers of water‐dependent ecosystems (Poff et al., 1997; Walker et al., 1995), variables other than hydrology also drive ecological structure and function (Poff et al., 2010; Tracy et al., 2024), particularly across spatial and temporal scales (Thompson et al., 2018). For example, Tracy et al. (2024) identified heat stress, physical damage (e.g., due to storms), and moisture deficit as the stressors that were most likely to cause mortality in a restoration experiment in riparian vegetation, while precipitation and altitude were key in identifying suitable areas for Siberian cranes (Liu et al., 2023). Such dependence on multiple factors underlies ideas of the niche as an n‐dimensional hypervolume (Hutchinson, 1957) and environmental filters (Kraft et al., 2015; Poff, 1997), and this is why most species distribution models correlate species presence with several environmental factors (Elith & Leathwick, 2009). Thus, hydrologic variables such as inundation can be a useful shorthand proxy for the conditions necessary to support a given species but may not be sufficient alone, and explicit consideration of other drivers of ecological processes is recommended (Arthington et al., 2018; Elith & Leathwick, 2009; Mentzafou et al., 2023). Our results clearly indicate that assessments based on hydrologic conditions in identified habitat types result in a very different outcome compared to an approach that considers biological processes, even when those processes are limited to those related to water. It is likely that the inclusion of temperature thresholds and other requirements would constrain favorable areas further or differently (Arthington et al., 2018).

Whether single or multiple, environmental variables rarely have simple linear relationships to biological outcomes. We see this in our process‐based models, where the plants do not respond to the presence of inundation per se, but to its timing and duration. In some situations, inundation is beneficial (initiating germination), while in others, it is detrimental (drowning seedlings or adults). While some species distribution models improve their handling of these issues using transformations of the underlying environmental variables (Elith & Leathwick, 2009), such transformations are limited in their ability to handle the sorts of dependencies addressed by process‐based models that explicitly describe their relationships to life‐stage success (Evans et al., 2015; Kearney & Porter, 2009).

One common solution to the issues arising from multiple environmental drivers and the complex biological responses to those drivers is to identify habitat types within which to restrict analyses. At the most general, this may mean examining only a particular continent or biome, as we have done here in restricting all analyses to wetlands. Such restrictions can be particularly informative for investigating contrasting constraints on species distributions in native and invaded areas or within historical ranges (e.g., Kuemmerle et al., 2011; Mainali et al., 2015). More specifically, habitat restrictions based on realized species presence incorporate unknown (and unmodeled) requirements without needing to explicitly identify all relevant environmental conditions (Holt et al., 2024), instead using the species themselves to identify locations that have been suitable over some recent time period. This approach also accentuates a key distinction between our questions and those of species distribution models. Management often focuses on short‐term (e.g., annual) reporting on favorability of conditions to monitor and assess risks and outcomes of management actions, rather than a long‐run prediction of species ranges (Hart et al., 2021; Mentzafou et al., 2023). Thus, while species distribution models attempt to predict ranges from environmental variables (Elith & Leathwick, 2009), our focus is on how changes in those variables affect favorability (not presence or absence), with the habitat types defining areas particularly relevant for this assessment.

Our habitat‐based models take two different approaches to such a delineation; the named wetland types get their names from the dominant vegetation type within each wetland (Brooks, 2021), while the recorded wetland types capture all types where species have been recorded. These definitions yield striking differences in estimated favorability, particularly when combined with process‐based models. Recruitment shows much less congruence with named types than recorded. A naïve expectation might be that the wetlands dominated by a species are a good indicator of favorable locations. However, all wetlands contain many species, and so using only named wetland types risks missing large areas of the range where species are present but not dominant. While all species have significant numbers of observations outside their named types, these discrepancies are most striking for lignum, which has the highest congruence of any species between recruitment and recorded wetland types, and yet almost no recruitment within named wetland types. Not only does the choice of habitat‐based, process‐based, or combined models have a large effect, so too does the choice of habitat definition.

Comparing the combined models to the habitat‐ and process‐based models alone helps identify the consequences of the various model choices. Moving from habitat‐based models to combined models requiring favorability for life‐cycle processes within that habitat shows that capturing complex relationships between the environment and ecology, rather than using inundation as a simple proxy, reduces the outcome by several orders of magnitude. Life‐cycle requirements are much more stringent than the existence of inundation. In reverse, imposing habitat‐based requirements on a process model is common practice and has benefits such as accounting for unmodeled relationships, but this approach is static and risks losing areas that may become favorable under changed conditions or where the original habitat assessment is limited. In many years, we find large areas favorable for the processes that are outside of the identified habitats, indicating potential opportunities for population expansion. Taken together, these results demonstrate that the selected methodology has huge implications for the conclusions drawn from the assessment—never an ideal circumstance.

The various life stages considered here (adults, regeneration, and recruitment) had very different amounts of favorable area identified, and the areas that were favorable for one life stage were not always favorable for another. The processes that we modeled are a subset of requirements for the maintenance of viable populations. Other life stages exist, and other thresholds likely limit various life‐history events, so reality will be far more nuanced than presented here. For example, competition for light is common among many *Eucalypt* species, with disturbance often required for recruitment of mature adults into the canopy (Bailey et al., 2012). Nonetheless, we attempted to identify the primary requirements listed in major works for woody vegetation in the Murray–Darling Basin (Roberts & Marston, 2011; Rogers & Ralph, 2010) and those for which there is the best information available (see references in Table 1). Importantly, the requirements captured here result in large differences between life stages and species. For example, favorable conditions for adult survival were common, but seedling establishment was the limiting factor for all species modeled. This is consistent with seedling establishment being recognized as the critical life stage for trees such as river red gum (Roberts & Marston, 2011). The requirements for long durations of soil moisture tended to be limiting, illustrated by the contrast between coolabah and lignum. These species have no inundation limitation and the strictest inundation requirements, respectively, and yet coolabah has only one successful recruitment event, while lignum shows the largest and most frequent recruitment events due to the short duration of its soil moisture requirement. Investigation of wider intervals of acceptable soil moisture (e.g., from the 10%–30% range used here to 5%–60%) yielded little change in the overall pattern (not shown), though using a biologically unreasonable lower limit of zero would make this stricture irrelevant. The recruitment patterns seen here are therefore likely to be biologically realistic, despite the challenges of assessing survival with the available inundation layer, though a better empirical understanding of the soil moisture requirements would improve the estimates. Furthermore, it is also likely to be realistic that conditions favoring adult survival, for example, may be very different from those favoring seedling establishment, given differences in the maximum duration of inundation each stage can withstand and the greater dependence of seedlings on continuous shallow soil moisture due to smaller root systems (Johns et al., 2009; Rogers & Ralph, 2010). Thus, while additional research would provide better process models, the responses seen here are striking, and the fundamental messages are unlikely to change as a more nuanced understanding of biological processes develops, at least in terms of the relative differences between habitat‐based and process‐based assessments.

It is important to note that persistence does not require all life stages to be successful in a single location in a single year, and for long‐lived species such as our floodplain trees, some life stages occur very rarely. Records of coolabah recruitment, for example, can be as infrequent as six times per century, while red gum also recruits infrequently (Ngugi et al., 2022; Roberts & Marston, 2011). River red gum has a potential life span of up to 100–950 years, noting that reliable estimates are rare (Roberts & Marston, 2011), so infrequent recruitment is unlikely to pose a meaningful limitation on healthy populations. Estimating such infrequent events creates challenges with even relatively long time series. Here, our data spanned 1988–2022 but, due to the long dependence of adults on previous inundation, the full life cycle could only be assessed from 2008. Based on the historical rate of six recruitment events per century, we would expect, on average, to have one favorable coolabah recruitment event. This matches the finding from our process model, though we should not surmise based on two uncertain values. Instead, the key message is that the low amount of favorable area for recruitment is likely a realistic assessment of natural processes, rather than a harbinger of doom for the species.

One approach to dealing with the differential frequency of favorability for different life‐stage events is to explicitly consider populations that spend most of their time in a maintenance phase, with intermittent expansion phases. Indeed, many plant species are characterized by resistant long‐lived life stages that maintain stable population sizes over long times, with rare recruitment events (e.g., Condit et al., 2017; Ngugi et al., 2022; Pierson & Turner, 1998). For our species, adult survival is likely a reasonable choice to drive the maintenance phase—that is, enable extant populations to persist through time. The amount of area identified as favorable for adults (e.g., Figure 6b) exceeded the area identified as favorable based on habitat types for all of our four species. This is in stark contrast to the amount of area favorable for recruitment, which drives population expansion. Recruitment is highly localized in space and time but this may be sufficient over large time and space to enable a self‐sustaining population, dependent on the balance with slow attrition from the maintenance phase. Both the long persistence of the maintenance phase and the need for infrequent expansion are common features of population dynamics models (e.g., Silvertown et al., 1993; Van Mantgem & Stephenson, 2005), but raise important questions about how long‐term favorability should be translated to an annual metric, which is far more commonly used for management purposes (Hart et al., 2021; Mentzafou et al., 2023).

There are opportunities associated with explicitly considering a modeling approach that combines habitat‐based and process‐based methods and then identifying the differences between the component parts. The differences between models can be helpful in uncovering why predictions differ or to quantify risk (Dormann et al., 2012; Elith & Leathwick, 2009; Kearney & Porter, 2009). For example, it may be possible to draw ecological inference from the differences in favorable area identified in space and time. Areas that are identified as favorable based on known habitats but not key processes may indicate areas of possible senescence. For long‐lived species such as woody vegetation, it can take decades before a lack of successful recruitment becomes apparent, risking the loss of existing populations. In such a case, the differences in area identified between modeling approaches could provide an opportunity to improve our understanding of spatial variability and non‐stationarity (Arthington et al., 2018). Conversely, areas that are identified as favorable based on processes that are outside the historical range or existing habitats of a species may be areas of possible range expansion, assuming that dispersal and establishment are possible (MacLean & Beissinger, 2017). Each of these interpretations would need to be based on confidence that the existing habitat delineation was correct, and that the critical processes had been identified, included, and accurately represented. Each is challenging in some circumstances, given the patchy and incomplete knowledge of ecology that has led to the use of habitat‐based proxies in the first place (Poff et al., 2010). Thus, we recommend care in the interpretation of differences between models—identifying areas of possible senescence or expansion—but then further exploration of that likelihood is required.

Emerging and linked modeling approaches may provide new, more robust pathways. Species distribution models, for example, can be linked with landscape, population, and physiological models that may provide a better representation of changing processes (Dormann et al., 2012; Elith & Leathwick, 2009; Kearney & Porter, 2009). In concept, this is what we have done here with our process‐based models, which link the underlying environmental drivers to simple population models that capture the physiological requirements of the life stages. Though our focus is on yearly favorability, we encounter common issues linking processes with species distribution models, with limited availability of detailed physiological requirements as well as data on a fine enough scale to capture them (Dormann et al., 2012). Even so, the models yield dramatic differences, identifying not only the importance of method choice but also areas needing further investigation (Kearney & Porter, 2009). New methods, such as the asymptotic environmentally determined trajectory (aedt), have been explicitly developed to incorporate the effect of life history on environmental responses, thereby incorporating lags and non‐stationarity effects (Chesson et al., 2024). However, the ability to apply these methods relies on the availability of appropriate data and knowledge; for example, aedt modeling cannot be used with a habitat‐only approach, as it depends on the dynamical properties of the population.

Any comprehensive effort to address the issues identified here requires a substantial re‐focus for ecology and ecohydrology. Fundamentally, new knowledge is needed on large‐scale processes that can be generalized in space and time. Critical questions include the following: Which thresholds are critical for survival for key species and communities? Which life‐history stages are limiting? How do these relationships vary in space and time and what other variables do they interact with? How can we efficiently gather information regarding processes about which we currently know little (e.g., seedling survival here)? What drivers need to be included that are not water related, among others. Such a shift, from our current general knowledge (Arthington et al., 2018; Poff et al., 2010) to specific knowledge at scale, is a huge undertaking that will likely be many years in the making. However, the current all‐too‐frequent focus on single species in a small number of locations and at a small number of times (Olden et al., 2014) is not sufficient to tackle the types and scale of questions that natural resource managers face, resulting in the types of simplifications tested here. As we have shown here, these simplifications have consequences, which may not be readily apparent and are rarely investigated in use. Synthesis attempts have been challenged by a lack of consistency in experimental design and reporting (Webb et al., 2013), indicating a large shift is needed. For ecology and ecohydrology to remain relevant in a rapidly changing world, we need a better understanding of how hydrologic systems and human demands are altering ecological communities at scale. To echo Arthington et al. (2018), our challenge as scientists is to build on existing knowledge to create new tools and approaches enabling managers to support diverse aquatic ecosystems in an uncertain future. As we demonstrate here, there is otherwise too much scope for uncertainty and error in the approaches that are commonly in use today.

## AUTHOR CONTRIBUTIONS

**Galen Holt:** Writing—review and editing; visualization; validation; software; methodology; investigation; formal analysis; data curation; conceptualization. **Georgia K. Dwyer:** Writing—review and editing; visualization; methodology; investigation; formal analysis; conceptualization. **Rebecca E. Lester:** Writing—original draft; supervision; investigation; conceptualization; funding acquisition.

## CONFLICT OF INTEREST STATEMENT

Galen Holt, Georgia K. Dwyer, and Rebecca E. Lester report financial support provided by Murray–Darling Basin Authority.

## Supporting information


Appendix S1:

## Data Availability

The Murray–Darling Basin Australian National Aquatic Ecosystem (ANAE) dataset (Brooks, 2021) is available from the Australian Department of Agriculture, Water and the Environment at https://fed.dcceew.gov.au/datasets/1e57385ab8374f51b4b518a8cf571dbc/. The two‐monthly inundation depth layer (Teng et al., 2023) is available in the Commonwealth Scientific and Industrial Research Organisation (CSIRO) Data Access Portal at https://doi.org/10.25919/c5ab-h019. Soil moisture data were downloaded from the Australian Government Bureau of Meteorology's Australian Water Outlook portal at https://awo.bom.gov.au/products/historical/soilMoisture-rootZone/ as NetCDF using the following data request: Product = Historical, Time aggregation = Day, Type = Relative, Variable = Root zone soil moisture, and Time = [each year 1980–2023]. Code (Holt & Dwyer, 2025) is available on Figshare at https://doi.org/10.6084/m9.figshare.27139446.v1.

## References

[eap70060-bib-0001] Arthington, A. H. , J. G. Kennen , E. D. Stein , and J. A. Webb . 2018. “Recent Advances in Environmental Flows Science and Water Management‐Innovation in the Anthropocene.” Freshwater Biology 63(8): 1022–1034. 10.1111/fwb.13108.

[eap70060-bib-0002] Bailey, T. G. , N. J. Davidson , and D. C. Close . 2012. “Understanding the Regeneration Niche: Microsite Attributes and Recruitment of Eucalypts in Dry Forests.” Forest Ecology and Management 269(April): 229–238. 10.1016/j.foreco.2011.12.021.

[eap70060-bib-0003] Belbin, L. , E. Wallis , D. Hobern , and A. Zerger . 2021. “The Atlas of Living Australia: History, Current State and Future Directions.” Biodiversity Data Journal 9(April): e65023. 10.3897/BDJ.9.e65023. 33935559 PMC8081701

[eap70060-bib-0004] Berezowski, T. , and M. Wassen . 2023. “Using Water Sources Extent during Inundation as a Reliable Predictor for Vegetation Zonation in a Natural Wetland Floodplain.” Ecological Indicators 154(October): 110854. 10.1016/j.ecolind.2023.110854.

[eap70060-bib-0005] Brooks, S. 2021. “ANAE Classification of the Murray‐Darling Basin Technical Report, Revision 3.0.” Technical Report for the Commonwealth Environmental Water Office, Department of Agriculture, Water and the Environment, Australia. https://fed.dcceew.gov.au/datasets/1e57385ab8374f51b4b518a8cf571dbc/.

[eap70060-bib-0006] Campbell, C. J. , S. Lovett , S. J. Capon , R. M. Thompson , and F. J. Dyer . 2023. “Beyond a ‘Just Add Water’ Perspective: Environmental Water Management for Vegetation Outcomes.” Journal of Environmental Management 348(December): 119499. 10.1016/j.jenvman.2023.119499. 37924694

[eap70060-bib-0007] Capon, S. J. , C. S. James , L. Williams , and G. P. Quinn . 2009. “Responses to Flooding and Drying in Seedlings of a Common Australian Desert Floodplain Shrub: *Muehlenbeckia florulenta* Meisn. (Tangled Lignum).” Environmental and Experimental Botany 66(2): 178–185.

[eap70060-bib-0008] Casanova, M. T. 2015. Review of Water Requirements for Key Floodplain Vegetation for the Northern Basin: Literature Review and Expert Knowledge Assessment. Lake Bolac: Murray‐Darling Basin Authority, Charophyte Services.

[eap70060-bib-0009] Chesson, P. , Y. J. Wu , and G. Holt . 2024. “The Asymptotic Environmentally Determined Trajectory (Aedt), Key to Understanding and Managing Ecological Systems under Climate Change.” Biological Conservation 293: 110526.

[eap70060-bib-0010] Condit, R. , R. Pérez , S. Lao , S. Aguilar , and S. P. Hubbell . 2017. “Demographic Trends and Climate over 35 Years in the Barro Colorado 50 ha Plot.” Forest Ecosystems 4(1): 1–13. 10.1186/s40663-017-0103-1.

[eap70060-bib-0011] Cowardin, L. M., V. Carter , F.C. Golet , and E.T. LaRoe . 1979. Classification of Wetlands and Deepwater Habitats of the United States. Washington D.C: Fish and Wildlife Service, US Department of the Interior.

[eap70060-bib-0012] Derepasko, D. , F. Witing , F. J. Peñas , J. Barquín , and M. Volk . 2023. “Towards Adaptive Water Management—Optimizing River Water Diversion at the Basin Scale under Future Environmental Conditions.” Water 15(18): 3289. 10.3390/w15183289.

[eap70060-bib-0013] Dormann, C. F. , S. J. Schymanski , J. Cabral , I. Chuine , C. Graham , F. Hartig , M. Kearney , et al. 2012. “Correlation and Process in Species Distribution Models: Bridging a Dichotomy.” Journal of Biogeography 39(12): 2119–2131. 10.1111/j.1365-2699.2011.02659.x.

[eap70060-bib-0014] Durant, R. , F. Freestone , D. Linklater , C. Reid , and C. Campbell . 2020. “Recruitment of Long‐Lived Floodplain Vegetation: Literature Review.” https://opal.latrobe.edu.au/articles/journal_contribution/Recruitment_of_long‐lived_floodplain_vegetation_Literature_review/8947394/files/16353830.pdf.

[eap70060-bib-0015] Elith, J. , and J. R. Leathwick . 2009. “Species Distribution Models: Ecological Explanation and Prediction across Space and Time.” Annual Review of Ecology, Evolution, and Systematics 40(1): 677–697. 10.1146/annurev.ecolsys.110308.120159.

[eap70060-bib-0016] Evans, T. G. , S. E. Diamond , and M. W. Kelly . 2015. “Mechanistic Species Distribution Modelling as a Link between Physiology and Conservation.” Conservation Physiology 3(1): cov056. 10.1093/conphys/cov056. 27293739 PMC4778482

[eap70060-bib-0017] Frost, A. J. , A. Ramchurn , and A. Smith . 2018. “The Australian Landscape Water Balance Model (AWRA‐L V6).” Technical Description of the Australian Water Resources Assessment Landscape Model Version 6. https://awo.bom.gov.au/products/historifcal/soilMoisture-rootZone/.

[eap70060-bib-0018] Gebreegziabher, G. A. , S. Degefa , W. Furi , and G. Legesse . 2023. “Evolution and Concept of Environmental Flows (e‐Flows): Meta‐Analysis.” Water Supply 23(6): 2466–2490. 10.2166/ws.2023.120.

[eap70060-bib-0019] Greet, J. , S. Fischer , C. J. Walsh , M. J. Sammonds , and J. A. Catford . 2022. “Restored River‐Floodplain Connectivity Promotes Riparian Tree Maintenance and Recruitment.” Forest Ecology and Management 506(February): 119952. 10.1016/j.foreco.2021.119952.

[eap70060-bib-0020] Hart, B. T. , N. R. Bond , N. Byron , C. A. Pollino , and M. J. Stewardson . 2021. “Chapter 1 – Introduction to the Murray–Darling Basin System, Australia.” In Murray‐Darling Basin, Australia, Vol. 1, edited by B. T. Hart , N. R. Bond , N. Byron , C. A. Pollino , and M. J. Stewardson , 1–17. Ecohydrology from Catchment to Coast. Oxford: Elsevier. 10.1016/B978-0-12-818152-2.00001-2.

[eap70060-bib-0021] Holt, G. , and G. Dwyer . 2025. “Code for Method Matters: Comparing Habitat‐ and Process‐Based Approaches for Favourability Assessment.” Figshare. 10.6084/m9.figshare.27139446.v1. PMC12147063 40485621

[eap70060-bib-0022] Holt, G. , A. Macqueen , and R. E. Lester . 2024. “A Flexible Consistent Framework for Modelling Multiple Interacting Environmental Responses to Management in Space and Time.” Journal of Environmental Management 367: 122054. 39106797 10.1016/j.jenvman.2024.122054

[eap70060-bib-0023] Hutchinson, G. E. 1957. “Concluding Remarks.” Cold Spring Harbor Symposia on Quantitative Biology 22(January): 415–427.

[eap70060-bib-0024] Jensen, A. E. 2008. “The Roles of Seed Banks and Soil Moisture in Recruitment of Semi‐Arid Floodplain Plants: The River Murray, Australia.”

[eap70060-bib-0025] Johns, C. , C. J. Reid , J. Roberts , N. Sims , T. Doody , I. Overton , H. McGinness , K. Rogers , C. Campbell , and B. Gawne . 2009. “Literature Review and Identification of Research Priorities to Address Retaining Floodwater on Floodplains and Flow Enhancement Hypotheses Relevant to Native Tree Species.” Report Prepared for the Murray‐Darling Basin Authority by The Murray‐Darling Freshwater Research Centre, June. 70 pp.

[eap70060-bib-0026] Kearney, M. , and W. Porter . 2009. “Mechanistic Niche Modelling: Combining Physiological and Spatial Data to Predict Species' Ranges.” Ecology Letters 12(4): 334–350. 10.1111/j.1461-0248.2008.01277.x. 19292794

[eap70060-bib-0027] Kraft, N. J. B. , P. B. Adler , O. Godoy , and E. C. James . 2015. “Community Assembly, Coexistence and the Environmental Filtering Metaphor.” Functional Ecology 29: 592–599. 10.1111/1365-2435.12345.

[eap70060-bib-0028] Kuemmerle, T. , V. C. Radeloff , K. Perzanowski , P. Kozlo , T. Sipko , P. Khoyetskyy , A.‐T. Bashta , et al. 2011. “Predicting Potential European Bison Habitat across Its Former Range.” Ecological Applications 21(3): 830–843. 10.1890/10-0073.1. 21639048

[eap70060-bib-0029] Leone, M. , F. Gentile , A. Lo Porto , G. F. Ricci , C. Schürz , M. Strauch , M. Volk , and A. M. De Girolamo . 2024. “Setting an Environmental Flow Regime under Climate Change in a Data‐Limited Mediterranean Basin with Temporary River.” Journal of Hydrology: Regional Studies 52(April): 101698. 10.1016/j.ejrh.2024.101698.

[eap70060-bib-0030] Lester, R. E. , H. M. McGinness , A. E. Price , A. Macqueen , N. L. R. Poff , and B. Gawne . 2020. “Identifying Multiple Factors Limiting Long‐Term Success in Environmental Watering.” Marine and Freshwater Research 71(2): 238–254.

[eap70060-bib-0031] Liu, X. , Z. Zhang , J. Zhang , B. Zhu , and J. Tian . 2023. “Projection of the Potential Distribution of Suitable Habitats for Siberian Crane (*Grus leucogeranus*) in the Middle and Lower Reaches of the Yangtze River Basin.” Frontiers in Earth Science 11: 1193677. 10.3389/feart.2023.1193677.

[eap70060-bib-0032] MacLean, S. A. , and S. R. Beissinger . 2017. “Species' Traits as Predictors of Range Shifts under Contemporary Climate Change: A Review and Meta‐Analysis.” Global Change Biology 23(10): 4094–4105. 28449200 10.1111/gcb.13736

[eap70060-bib-0033] Mainali, K. P. , D. L. Warren , K. Dhileepan , A. McConnachie , L. Strathie , G. Hassan , D. Karki , B. B. Shrestha , and C. Parmesan . 2015. “Projecting Future Expansion of Invasive Species: Comparing and Improving Methodologies for Species Distribution Modeling.” Global Change Biology 21(12): 4464–4480. 10.1111/gcb.13038. 26185104

[eap70060-bib-0034] Mentzafou, A. , P. Katsafados , A. Papadopoulos , and E. Dimitriou . 2023. “Hydrological Regime Alteration Assessment in the Context of WFD 2000/60: A European and Global Review.” Sustainability 15(22): 15704. 10.3390/su152215704.

[eap70060-bib-0035] Ngugi, M. R. , V. J. Neldner , R. M. Dowling , and J. Li . 2022. “Recruitment and Demographic Structure of Floodplain Tree Species in the Queensland Murray‐Darling Basin, Australia.” Ecological Management & Restoration 23(1): 64–73. 10.1111/emr.12525.

[eap70060-bib-0036] Olden, J. D. , C. P. Konrad , T. S. Melis , M. J. Kennard , M. C. Freeman , M. C. Mims , E. N. Bray , K. B. Gido , N. P. Hemphill , and D. A. Lytle . 2014. “Are Large‐Scale Flow Experiments Informing the Science and Management of Freshwater Ecosystems?” Frontiers in Ecology and the Environment 12(3): 176–185.

[eap70060-bib-0037] Overton, I. C. , C. A. Pollino , J. Roberts , J. R. W. Reid , N. R. Bond , H. M. McGinness , B. Gawne , D. S. Stratford , L. E. Merrin , and D. Barma . 2014. “Development of the Murray‐Darling Basin Plan SDL Adjustment Ecological Elements Method.” Report Prepared by CSIRO for the Murray‐Darling Basin Authority, Canberra, 45–54.

[eap70060-bib-0038] Palmer, M. A. , H. L. Menninger , and E. Bernhardt . 2010. “River Restoration, Habitat Heterogeneity and Biodiversity: A Failure of Theory or Practice?” Freshwater Biology 55: 205–222.

[eap70060-bib-0039] Pierson, E. A. , and R. M. Turner . 1998. “An 85‐Year Study of Saguaro (*Carnegiea gigantea*) Demography.” Ecology 79(8): 2676–2693. 10.1890/0012-9658(1998)079[2676:AYSOSC]2.0.CO;2.

[eap70060-bib-0040] Poff, N. L. 1997. “Landscape Filters and Species Traits: Towards Mechanistic Understanding and Prediction in Stream Ecology.” Journal of the North American Benthological Society 16(2): 391–409 http://www.jstor.org.ezproxy1.library.arizona.edu/stable/1468026.

[eap70060-bib-0041] Poff, N. L. , J. D. Allan , M. B. Bain , J. R. Karr , K. L. Prestegaard , B. D. Richter , R. E. Sparks , and J. C. Stromberg . 1997. “The Natural Flow Regime.” BioScience 47(11): 769–784. http://www.jstor.org.ezproxy1.library.arizona.edu/stable/1313099.

[eap70060-bib-0042] Poff, N. L. , B. D. Richter , A. H. Arthington , S. E. Bunn , R. J. Naiman , E. Kendy , M. Acreman , et al. 2010. “The Ecological Limits of Hydrologic Alteration (ELOHA): A New Framework for Developing Regional Environmental Flow Standards.” Freshwater Biology 55(1): 147–170. 10.1111/j.1365-2427.2009.02204.x.

[eap70060-bib-0043] Railsback, S. F. 2016. “Why It Is Time to Put PHABSIM Out to Pasture.” Fisheries 41(12): 720–725.

[eap70060-bib-0044] Roberts, J. , and F. Marston . 2011. Water Regime for Wetland and Floodplain Plants: A Source Book for the Murray‐Darling Basin. Canberra: National Water Commission.

[eap70060-bib-0045] Rogers, K. , and T. J. Ralph . 2010. Floodplain Wetland Biota in the Murray‐Darling Basin: Water and Habitat Requirements. Collingwood: CSIRO Publishing.

[eap70060-bib-0046] Rosenfeld, J. S. 2017. “Developing Flow‐Ecology Relationships: Implications of Nonlinear Biological Responses for Water Management.” Freshwater Biology 62(8): 1305–1324. 10.1111/fwb.12948.

[eap70060-bib-0047] Ryder, D. S. , M. Tomlinson , B. Gawne , and G. E. Likens . 2010. “Defining and Using ‘Best Available Science’: A Policy Conundrum for the Management of Aquatic Ecosystems.” Marine and Freshwater Research 61(7): 821–828.

[eap70060-bib-0048] Silvertown, J. , M. Franco , I. Pisanty , and A. Mendoza . 1993. “Comparative Plant Demography—Relative Importance of Life‐Cycle Components to the Finite Rate of Increase in Woody and Herbaceous Perennials.” Journal of Ecology 81(3): 465–476. 10.2307/2261525.

[eap70060-bib-0049] Sutherland, W. J. 2006. “Predicting the Ecological Consequences of Environmental Change: A Review of the Methods.” Journal of Applied Ecology 43: 599–616.

[eap70060-bib-0050] Teng, J. , D. Penton , C. Ticehurst , A. Sengupta , A. Freebairn , S. Marvanek , D. King , and C. A. Pollino . 2023. “Two‐Monthly Maximum Flood Water Depth Spatial Timeseries for the MDB V2.” CSIRO Data Collection. 10.25919/c5ab-h019. PMC10517945 37741870

[eap70060-bib-0051] Tharme, R. E. 2003. “A Global Perspective on Environmental Flow Assessment: Emerging Trends in the Development and Application of Environmental Flow Methodologies for Rivers.” River Research and Applications 19(5–6): 397–441. 10.1002/rra.736.

[eap70060-bib-0052] Thompson, R. M. , A. J. King , R. M. Kingsford , R. Mac Nally , and N. L. R. Poff . 2018. “Legacies, Lags and Long‐Term Trends: Effective Flow Restoration in a Changed and Changing World.” Freshwater Biology 63(8): 986–995.

[eap70060-bib-0053] Tracy, J. E. , A. Sharma , M. Deitch , J. Colee , M. Thetford , and D. Johnson . 2024. “Flood Dynamics and Tree Resilience: First‐Year Seedlings of Five Floodplain Forest Species Responding to Diverse Inundation Scenarios.” Forest Ecology and Management 556(March): 121724. 10.1016/j.foreco.2024.121724.

[eap70060-bib-0054] Van Appledorn, M. , N. R. De Jager , and J. J. Rohweder . 2024. “Low‐Complexity Floodplain Inundation Model Performs Well for Ecological and Management Applications in a Large River Ecosystem.” Journal of the American Water Resources Association 60(1): 9–26. 10.1111/1752-1688.13152.

[eap70060-bib-0055] Van Mantgem, P. J. , and N. L. Stephenson . 2005. “The Accuracy of Matrix Population Model Projections for Coniferous Trees in the Sierra Nevada, California.” Journal of Ecology 93(4): 737–747. 10.1111/j.1365-2745.2005.01007.x.

[eap70060-bib-0056] Vaughan, I. P. , M. Diamond , A. M. Gurnell , K. A. Hall , A. Jenkins , N. J. Milner , L. A. Naylor , D. A. Sear , G. Woodward , and S. J. Ormerod . 2009. “Integrating Ecology with Hydromorphology: A Priority for River Science and Management.” Aquatic Conservation: Marine and Freshwater Ecosystems 19(1): 113–125.

[eap70060-bib-0057] Walker, K. F. , F. Sheldon , and J. T. Puckridge . 1995. “A Perspective on Dryland River Ecosystems.” Regulated Rivers: Research & Management 11(1): 85–104.

[eap70060-bib-0058] Webb, A. J. , K. A. Miller , E. L. King , S. C. de Little , M. J. Stewardson , J. K. H. Zimmerman , and N. L. R. Poff . 2013. “Squeezing the Most Out of Existing Literature: A Systematic Re‐Analysis of Published Evidence on Ecological Responses to Altered Flows.” Freshwater Biology 58(12): 2439–2451. 10.1111/fwb.12234.

[eap70060-bib-0059] Weiskopf, S. R. , M. A. Rubenstein , L. G. Crozier , S. Gaichas , R. Griffis , J. E. Halofsky , K. J. W. Hyde , et al. 2020. “Climate Change Effects on Biodiversity, Ecosystems, Ecosystem Services, and Natural Resource Management in the United States.” Science of the Total Environment 733(September): 137782. 10.1016/j.scitotenv.2020.137782. 32209235

[eap70060-bib-0060] Westgate, M. , M. Stevenson , D. Kellie , and P. Newman . 2024. “galah: Biodiversity Data from the GBIF Node Network.” https://CRAN.R-project.org/package=galah.

