# Supplementary material for: Method matters: Comparing habitat‐ and process‐based approaches for favorability assessment
Source: Ecol Appl. 2025 Jun 9;35(4):e70060. doi: 10.1002/eap.70060 (PMC12147063; doi:10.1002/eap.70060)
Supplement: Supplementary file 1 — Appendix S1: [file EAP-35-e70060-s001.pdf]

Method matters: Comparing habitat- and process-based approaches for favorability assessment

Appendix S1

Ecological Applications

Galen Holt, Georgia K Dwyer, and Rebecca E Lester

Wetland types

The number of ALA (Atlas of Living Australia) records was highly variable across wetland type and varies between species (Table S1). Strikingly, the named wetland types were not always the wetland types with the most records and, in some cases, were even excluded from the recorded set because they contained < 0.5% of the total records.

Table S1. Habitat types with records in Atlas of Living Australia for each of the four target species, listing the number of records. ANAE (Australian National Aquatic Ecosystem classification) Wetland Types is the name of the wetland type from the ANAE ("ANAE_DESC"; Brooks, 2021). Bold cells with green background are the named wetland types (with the species name in the type description). Those in light grey are < 0.5% of the total and were not included in the set of ANAE wetland types used. Note that for all species, there are `recorded` wetland types that have more records than some `named` types. This is particularly striking for lignum, where the type with the most records is black box woodlands. In some cases (black box and coolabah), there are named wetland types with < 0.5% of the records, and so these are not included in `recorded` types. Empty cells are wetland types with no Atlas of Living Australia records (zeros are not used because the ALA returns records, but does not reflect comprehensive surveys able to assess absence).

ANAE Wetland Type	Red gum	Black box	Coolabah	Lignum	Total records
River red gum forest riparian zone or floodplain	**9232**	663	185	907	10987
Black box woodland riparian zone or floodplain	1694	**7162**	296	1777	10929
River red gum woodland riparian zone or floodplain	**5063**	2237	10	651	7961
Coolabah woodland and forest riparian zone or floodplain	331	284	**2692**	1192	4499
Lignum shrubland riparian zone or floodplain	528	533	38	**895**	1994
Temporary river red gum swamp	**1482**	50	4	87	1623
Temporary lowland stream	754	234	106	270	1364
Permanent lake	787	51	8	360	1206
Shrubland riparian zone or floodplain	344	491	9	269	1113
Temporary swamps	690	131		93	914
Permanent lowland stream	553	72	27	109	761
Freshwater meadow	286	92	115	263	756
Temporary tall emergent marsh	383	85		195	663
Temporary lake	275	106	11	121	513
Permanent wetland	285	20	8	83	396
Unspecified riparian zone or floodplain	239	58		80	377
Temporary black box swamp	73	**167**	3	128	371
Clay pan	152	72	1	115	340
Woodland riparian zone or floodplain	188	51	29	45	313
Temporary sedge/grass/forb marsh	95	9	6	137	247
River cooba woodland riparian zone or floodplain	33	11	60	140	244
Temporary transitional zone stream	196	2	1	2	201

ANAE Wetland Type	Red gum	Black box	Coolabah	Lignum	Total records
Temporary shrub swamp	13	36		58	107
Temporary woodland swamp	32	26	12	32	102
Temporary lignum swamp	17	13		**61**	91
Temporary saline swamp	14	16		23	53
Black box forest riparian zone or floodplain	6	**17**		20	43
Permanent forb marsh	32			2	34
Sedge/forb/grassland riparian zone or floodplain	9	9		12	30
Permanent transitional zone stream	29				29
Temporary lake with aquatic bed	10	7		8	25
Temporary saline wetland	1	14		10	25
Permanent saline wetland	15	4		3	22
Permanent grass marsh	1			17	18
Temporary wetland	10	1	1	5	17
Temporary saline lake	6	7		1	14
Temporary stream	7		1	3	11
Permanent stream	4	1		3	8
Temporary coolabah swamp	2	2	**1**	3	8
Temporary sedge/grass/forb marsh	1			6	7
Tide-dominated saltmarsh				6	6
Permanent saline lake		4		1	5
Temporary low-energy upland stream	5				5
Permanent low-energy upland stream	3	1			4
Permanent tall emergent marsh	4				4
Temporary saline lake with aquatic bed	1	1		2	4
Tide-dominated forest				4	4
Temporary salt marsh	2			1	3
Tide-dominated estuary				3	3
Coastal lagoon				2	2
Permanent high-energy upland stream	2				2
Salt pan or salt flat	2				2
Temporary high-energy upland stream	2				2
Unspecified river	2				2
Tide-dominated mudflats and sandbar				1	1

The recorded wetland types were extracted from the ALA (Atlas of Living Australia; Belbin et al., 2021). Those records were intersected with ANAE polygons (all ANAE wetlands are shown in Figure S1) to determine the ANAE wetland types for which there were records of each of the four species. The records that intersect with ANAE wetlands are shown in green in Figure S2.

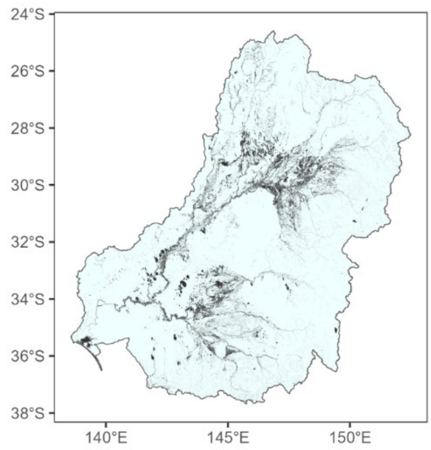

Figure S1: ANAE wetlands (grey) across the Murray-Darling Basin.

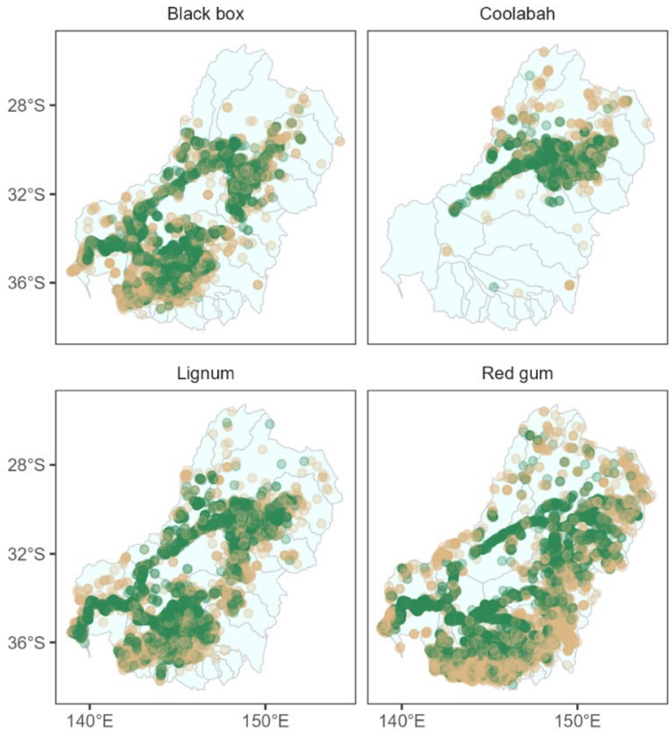

Figure S2: ALA records for each species that intersect with ANAE wetlands (green) or do not (brown). More than 73% of ALA records in the Murray-Darling Basin fall within ANAE wetlands for all species (black box 77%, red gum 74%, coolabah 74%, lignum 77%).

The area of wetlands in each wetland type was highly variable across the Murray-Darling Basin, with some species not having any named wetland types in entire catchments, or even nearly the entire southern Basin in the case of coolabah (Figure S2, Figure S3). Note that there are also Recorded habitat types in areas where there are no ALA records in ANAE wetlands (Figure S2).

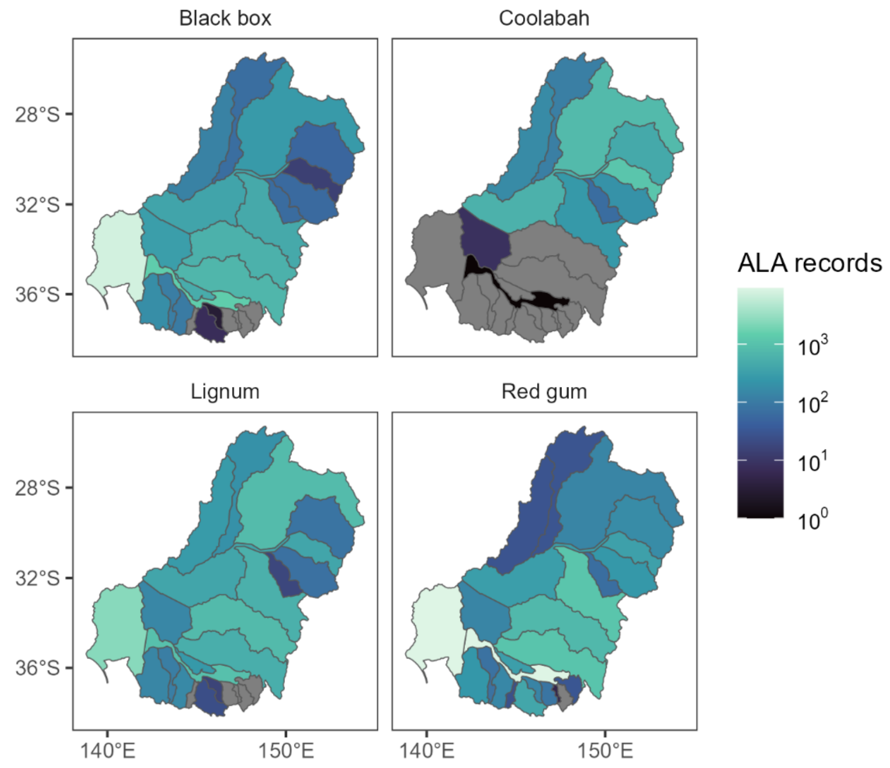

Figure S3. Number of ALA records within ANAE wetlands in each catchment for each species. Grey catchments have no ALA records for that species.

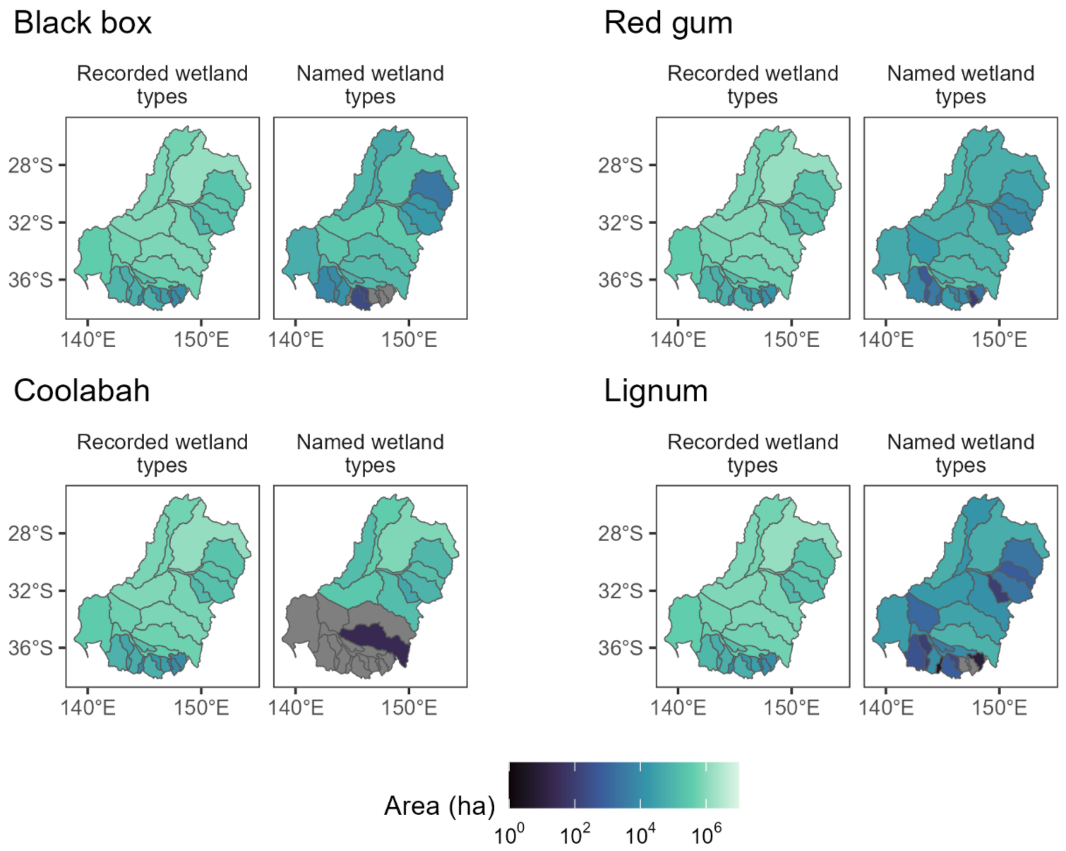

Figure S4. Area of wetlands associated with each species in 25 major catchments across the Murray-Darling Basin, either by Name or where that species has been Recorded in the ALA. This figure illustrates the total area of wetlands, and does not include any inundation information. Instead, it forms the baseline maximum amount of area that could be achieved within any grouping of wetland types within a catchment.

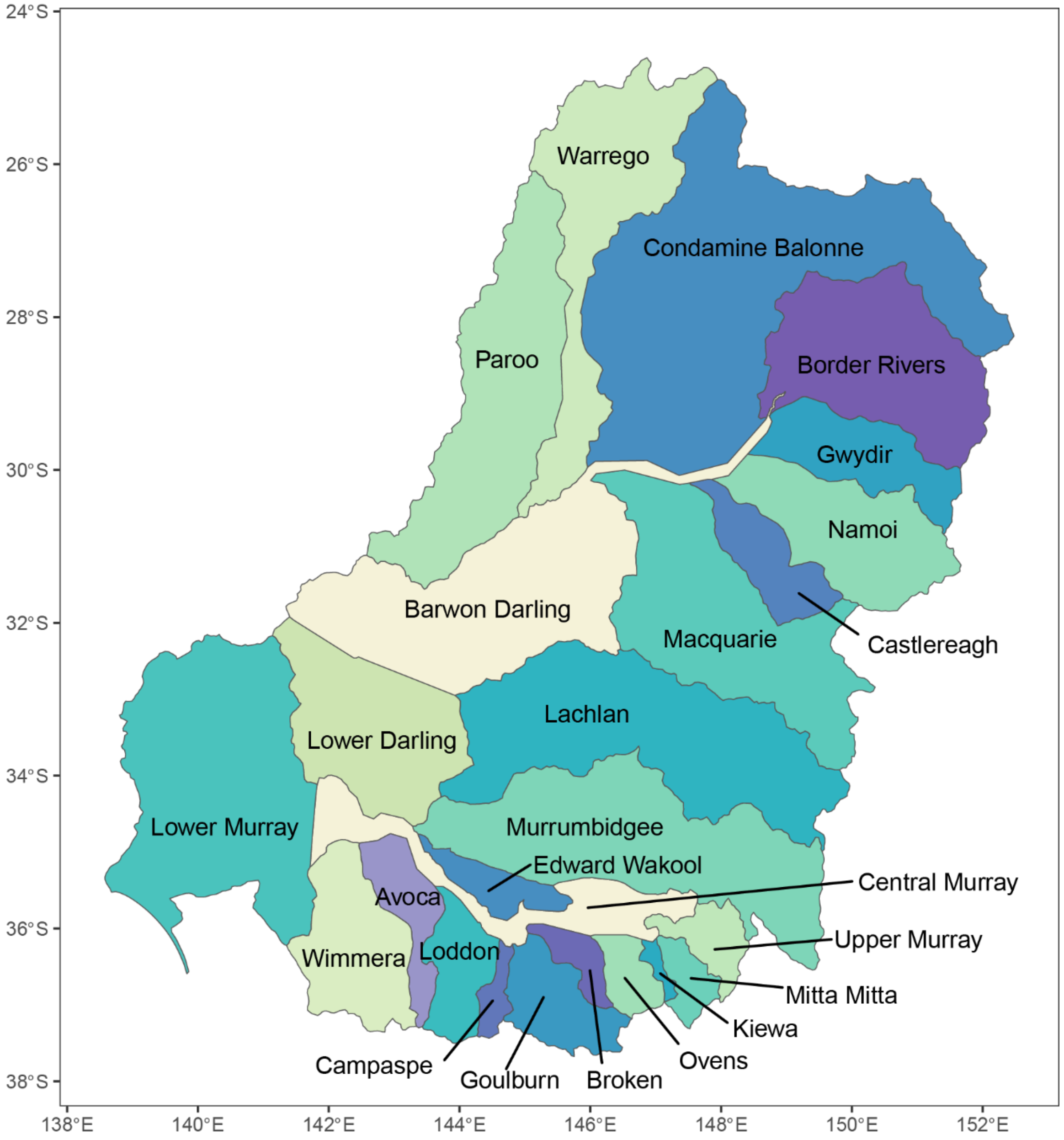

Figure S5. Catchments within the Murray-Darling Basin.

Figure 3 in the text shows all ANAE wetlands within the Riverland Wetland Complex Ramsar site in the Lower Murray catchment in South Australia, with location shown in Figure S6.

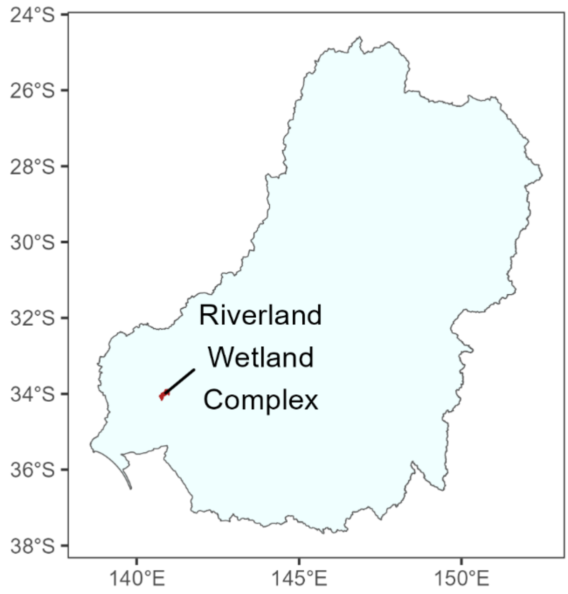

Figure S6. Location of Riverland Wetland Complex Ramsar site in Murray-Darling Basin. Figure 3 in the text shows all ANAE wetlands within this area.

Comparison of habitat-based and process-based models

The main text presents river red gum (*Eucalyptus camaldulensis*) as an example species. Here, we provide additional information about red gum, and also present the same figures and tables for three other species, noting important deviations from red gum. The three other species are black box (*Eucalyptus largiflorens*) (5), coolabah (*Eucalyptus coolabah*) (6) and lignum (*Duma florulenta*) (7). Refer to the main text for commentary regarding the characteristics of each species and the main differences among species.

When examined over space and time (i.e. the time average of the total favorable area in each catchment), each species shows dramatic differences in favorable area, no matter which model type is considered (Table S2 & Table S3). Within each species, the process-based and habitat-based models yield vast differences in the amount of favorable area, with their intersection (processes occurring within the correct wetland types; Recruitment and Recorded or Named and Recorded) having the lowest favorability (Table S2 & Table S3). To put this overlap in context, we also calculate the unique areas for each of the habitat- and process-based models, that is, the area of each type that does not spatially overlap favorable areas of the other (Table S2 & Table S3).

Table S2. Area of favorability (mean hectares over time in the Basin), with separated and combined categories for Recruitment (process-based model) and Recorded wetland types (habitat-based model). Grey shading columns are the full area of favorability in each model type, and are typically what are presented in the text and figures. Recruitment is the total area of recruitment favorability in any wetland type, Recorded wetland types is the total area of inundation in types with an ALA record, and Recruitment and Recorded is the area of Recorded wetland types in which Recruitment conditions were met. Unshaded columns are the areas *unique* to a model type, i.e. 'Recruitment outside Recorded' is (Recruitment – Recruitment and Recorded), and 'Recorded only' is (Recorded wetland types – Recruitment and Recorded).

	┌-----Recruitment-----┐	┌--------Recorded-------┐			
**Species**	**Recruitment outside Recorded**	**Recruitment and Recorded**	**Recorded only**	**Recruitment**	**Recorded wetland types**
Black box	9.01	0.61	223,130.50	9.62	268,510.08
Red gum	43.37	2.73	332,821.70	46.09	382,198.50
Coolabah	0.88	0.00	100,355.02	0.88	112,338.27
Lignum	79.31	63.95	349,198.86	143.26	410,092.86

Table S3. Area of favorability (mean hectares over time in the Basin), with separated and combined categories for Recruitment (process-based model) and Named wetland types (habitat-based model). Grey shading columns are the full area of favorability in each model type, and are typically what are presented in the text and figures. Recruitment is the total area of recruitment favorability in any wetland type, Named wetland types is the total area of inundation in types with an ALA record, and Recruitment and Named is the area of Named wetland types in which Recruitment conditions were met. Unshaded columns are the areas *unique* to a model type, i.e. 'Recruitment outside Named' is (Recruitment – Recruitment and Named), and 'Named only' is (Named wetland types – Recruitment and Named).

	┌--------Recruitment-------┐	┌--------Named-------┐			
**Species**	**Recruitment outside Named**	**Recruitment and Named**	**Named only**	**Recruitment**	**Named wetland types**
Black box	9.62	0.00	19,798.06	9.62	28,917.15
Red gum	45.96	0.14	39,285.43	46.09	45,161.23
Coolabah	0.88	0.00	40,888.74	0.88	47,159.51
Lignum	143.18	0.08	10,878.24	143.26	14,547.90

Basin-scale favorability

We present figures (Figure S6, S7, & S8) matching figure 4 in the text for each species, as well as tables matching Table 2 in the text (Table S4, S5, & S6), showing the area of favorability for the habitat- and process-based models, as well as their interaction for black box, coolabah, and lignum.

Black box

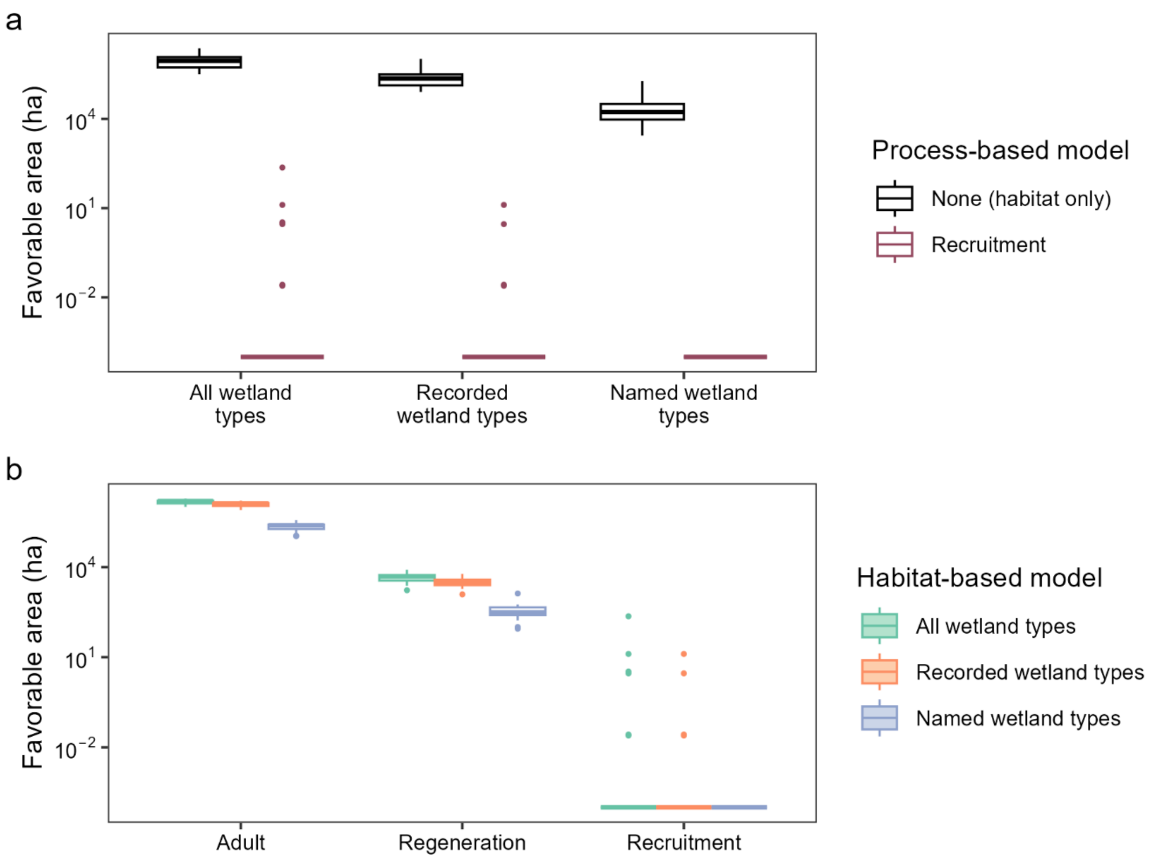

Figure S7. Favorable area for black box for habitat-based, process-based, and combined models at the basin scale. For further detail, see caption for Figure 4 in the text.

Table S4. Mean area (in hectares) over time in the Basin of black box favorability in habitat-based and process-based models. For further detail, see caption for Table 2 in the text.

	Habitat inundation	Adult	Regeneration	Recruitment
**All wetland types**	964,993.70	1,482,071.53	4,718.80	9.62
**Recorded wetland types**	268,510.08	1,231,578.43	3,311.86	0.61
**Named wetland types**	28,917.15	232,533.75	368.41	0.00

Coolabah

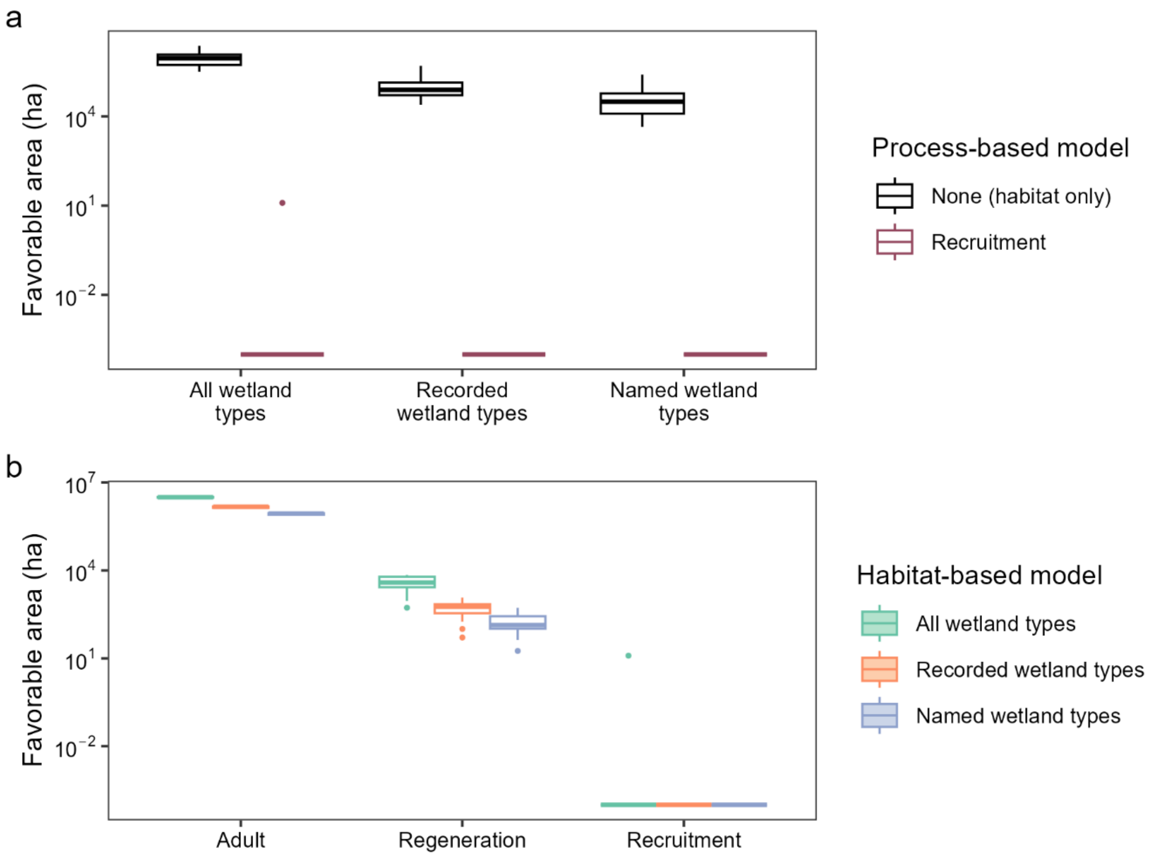

Figure S8. Favorable area for coolabah for habitat-based, process-based, and combined models at the basin scale. For further detail, see caption for Figure 4 in the text.

Table S5. Mean area (in hectares) over time in the Basin of coolabah favorability in habitat-based and process-based models. For further detail, see caption for Table 2 in the text.

	Habitat inundation	Adult	Regeneration	Recruitment
**All wetland types**	964,993.70	3,145,730.72	4,069.16	0.88
**Recorded wetland types**	112,338.27	1,460,685.35	529.56	0.00
**Named wetland types**	47,159.51	855,808.44	198.44	0.00

Lignum

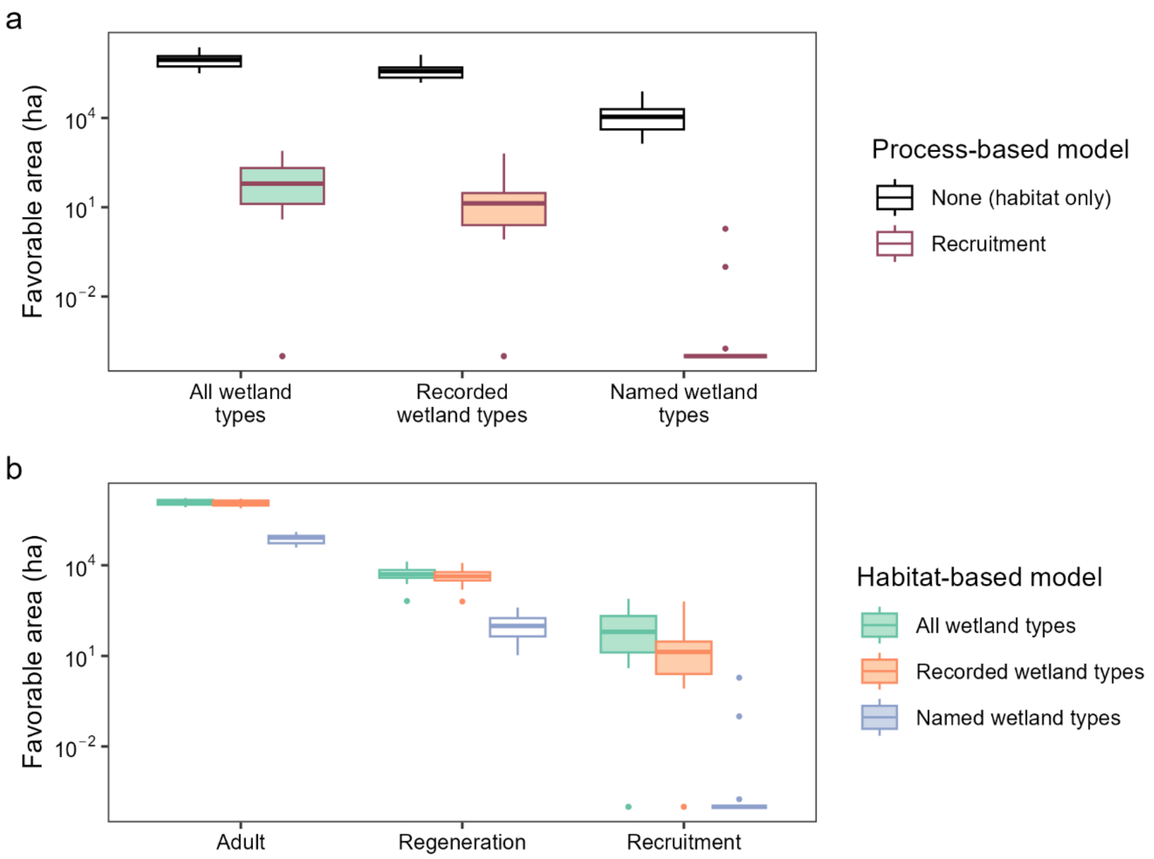

Figure S9. Favorable area for lignum for habitat-based, process-based, and combined models at the basin scale. For further detail, see caption for Figure 4 in the text.

Table S6. Mean area (in hectares) over time in the Basin of lignum favorability in habitat-based and process-based models. For further detail, see caption for Table 2 in the text.

	Habitat inundation	Adult	Regeneration	Recruitment
**All wetland types**	964,993.70	1,272,811.42	5,388.75	143.26
**Recorded wetland types**	410,092.86	1,200,982.19	4,643.91	63.95
**Named wetland types**	14,547.90	80,997.23	112.18	0.08

Favorability across space

Maps are shown here for all other species matching figure 5 in the text (Figure S9, S10, & S11). For ease of interpreting red gum results and discussion in the text, we include a table of favorable areas from each of the habitat- and process-based models, as well as their combinations (Table S7). We also include the loss of habitat-based models from inundation in all wetland types (Table S8), which is calculated from Table S7 but presented alone for clarity.

Table S7. Area of favorability for red gum in each catchment according to habitat-based, process-based, and combination models. All, Recorded, and Named wetland types assess favorability as inundation within the relevant set of wetlands, while Recruitment is favorability according to process requirements for Adults, Regeneration, and Seedling survival in sequence. Recruitment and Recorded and Recruitment and Named are the combination models, requiring those processes to occur within the relevant set of wetland types.

Catchment	All wetland types	Recorded wetland types	Named wetland types	Recruitment	Recruitment and Recorded	Recruitment and Named
Avoca	18,643.75	1,098.48	8.41	43.35	0.00	0.00
Barwon-Darling	114,259.74	55,226.70	5,707.94	0.09	0.09	0.00
Border Rivers	23,949.89	7,464.20	1,080.21	0.00	0.00	0.00
Broken	4,527.50	2,837.17	188.93	0.00	0.00	0.00
Campaspe	555.95	220.09	39.35	0.00	0.00	0.00
Castlereagh	6,678.14	1,735.77	245.48	0.00	0.00	0.00
Central Murray	69,073.70	35,641.59	11,105.3 0	0.17	0.16	0.13
Condamine-Balonne	119,598.77	34,059.44	1,077.81	0.00	0.00	0.00
Edward Wakool	12,280.67	5,308.29	1,788.40	0.00	0.00	0.00
Goulburn	10,369.98	5,403.24	1,676.15	0.01	0.01	0.01
Gwydir	14,116.92	4,050.92	372.89	0.00	0.00	0.00
Kiewa	482.90	0.00	56.13	0.00	0.00	0.00
Lachlan	53,588.30	22,731.82	2,270.81	0.00	0.00	0.00
Loddon	14,095.59	6,961.56	416.34	0.00	0.00	0.00
Lower Darling	51,269.93	27,431.23	3,164.53	0.00	0.00	0.00
Lower Murray	61,523.20	41,680.47	2,340.58	0.66	0.66	0.00
Macquarie	45,766.17	13,642.01	3,628.13	0.00	0.00	0.00
Mitta Mitta	771.50	0.00	6.45	0.00	0.00	0.00
Murrumbidgee	65,208.31	29,397.15	5,567.82	1.78	1.78	0.00
Namoi	27,409.44	12,026.91	505.19	0.03	0.03	0.00
Ovens	2,668.52	893.52	248.82	0.00	0.00	0.00
Paroo	176,954.00	51,201.76	1,405.04	0.00	0.00	0.00
Upper Murray	2,358.44	611.96	84.08	0.00	0.00	0.00
Warrego	47,382.12	14,072.21	1,700.43	0.00	0.00	0.00
Wimmera	21,460.29	8,502.01	476.03	0.00	0.00	0.00

Table S8. Loss in area of favorability for red gum for habitat-based models when moving from all wetland types to restricting inundation to Recorded types and Named types. Calculated as (area inundated in all types – area inundated in restricted types)/(area inundated in all types).

**Valley name**	**Recorded loss**	**Named loss**
Goulburn	0.48	0.84
Central Murray	0.48	0.84
Edward Wakool	0.57	0.85
Kiewa	1.00	0.88
Ovens	0.67	0.91
Murrumbidgee	0.55	0.91
Macquarie	0.70	0.92
Campaspe	0.60	0.93
Lower Darling	0.46	0.94
Barwon-Darling	0.52	0.95
Border Rivers	0.69	0.95
Lachlan	0.58	0.96
Broken	0.37	0.96
Lower Murray	0.32	0.96
Castlereagh	0.74	0.96
Warrego	0.70	0.96
Upper Murray	0.74	0.96
Loddon	0.51	0.97
Gwydir	0.71	0.97
Wimmera	0.60	0.98
Namoi	0.56	0.98
Condamine-Balonne	0.72	0.99
Mitta Mitta	1.00	0.99
Paroo	0.71	0.99
Avoca	0.94	1.00

Black box

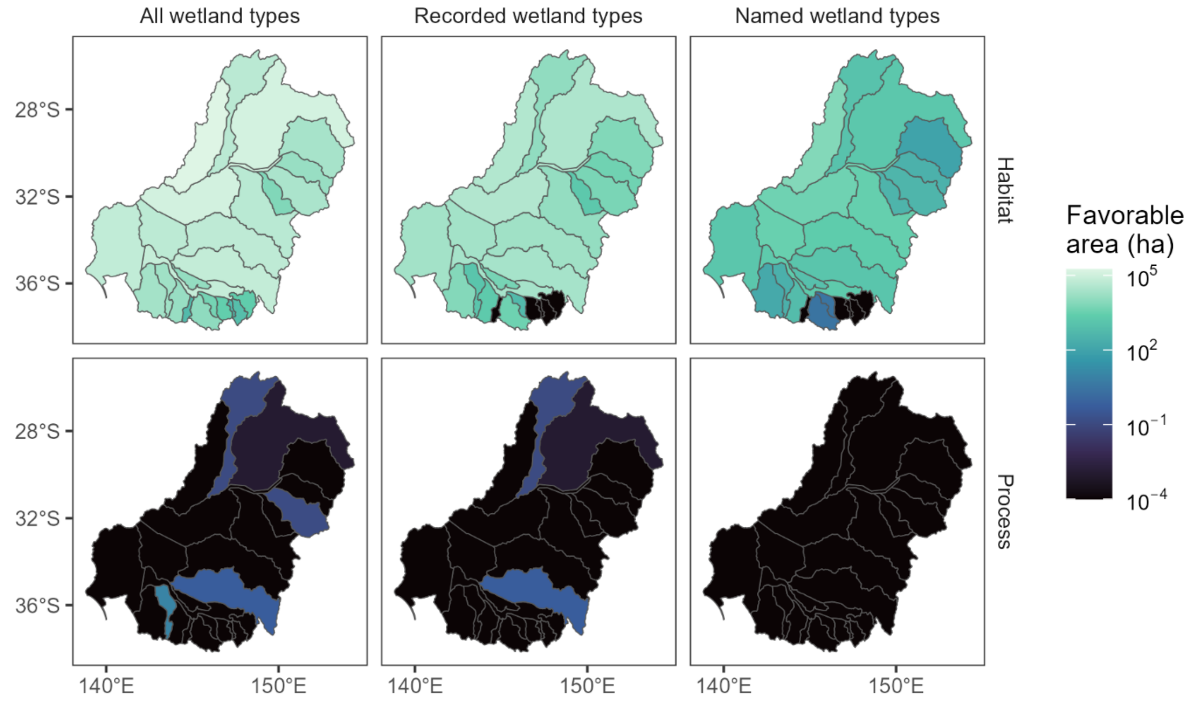

Figure S10. Spatial variation in the area identified as favorable for black box from habitat-based (top row), process-based (bottom left), and combined (bottom middle and right) models. For further detail see caption of Figure 5 in the text.

Coolabah

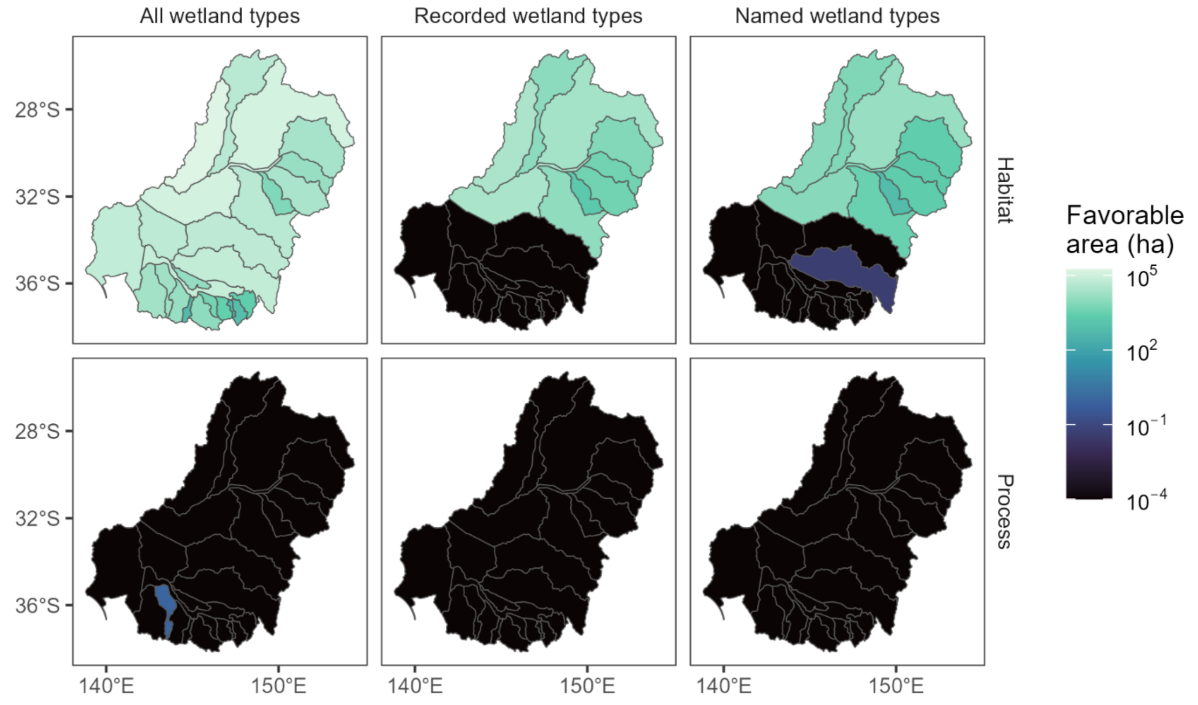

Figure S11. Spatial variation in the area identified as favorable for coolabah from habitat-based (top row), process-based (bottom left), and combined (bottom middle and right) models. For further detail see caption of Figure 5 in the text.

Lignum

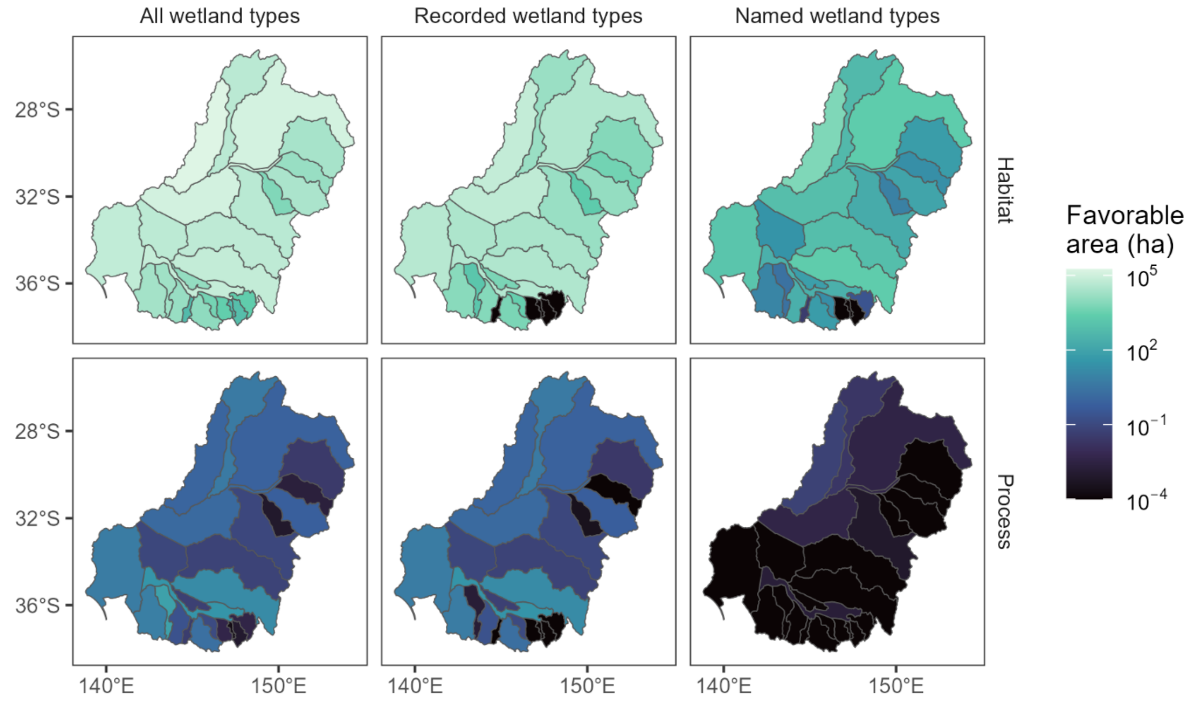

Figure S12. Spatial variation in the area identified as favorable for lignum from habitat-based (top row), process-based (bottom left), and combined (bottom middle and right) models. For further detail see caption of Figure 5 in the text.

Favorability through time

We show here the numerical values underlying Figure 6 in the text for red gum, along with columns for the unique values for each model type, i.e. the areas identified in habitat- or process-based models that did not overlap spatially with the other model type (Table S9). We also include timeseries figures paralleling figure 5 for the other three species (Figures S12, S13, & S14).

Table S9. Area of favorability (hectares) in each year for red gum, with separated and combined categories for Recruitment (process-based model) and Recorded wetland types (habitat-based model). Grey shading columns are the full area of favorability in each model type, and are presented in Figure 6 in the text. Recruitment is the total area of recruitment favorability in any wetland type, Recorded wetland types is the total area of inundation in types with an ALA record, and Recruitment and Recorded is the area of Recorded wetland types in which Recruitment conditions were met. Unshaded columns are the areas *unique* to a model type, i.e. 'Recruitment outside Recorded' is (Recruitment – Recruitment and Recorded), and 'Recorded only' is (Recorded wetland types – Recruitment and Recorded). Tables for other species not shown, see

Figures S12, S13, & S14. Empty cells for process models are due to extended lookback periods, mostly for adult favorability, and so cannot be assessed near the ends of the timeseries.

		┌-------Recruitment-----┐		┌----Recorded----┐		
**Species**	**Year**	**Recruitment outside Recorded**	**Recruitment and Recorded**	**Recorded only**	**Recruitment**	**Recorded wetland types**
Red gum	1988					465,668.32
Red gum	1989					707,941.46
Red gum	1990					652,981.66
Red gum	1991					358,201.55
Red gum	1992	0.00	0.00	392,720.71	0.00	392,720.71
Red gum	1993	0.00	0.00	408,884.52	0.00	408,884.52
Red gum	1994	0.33	0.51	273,140.24	0.83	273,141.07
Red gum	1995	0.00	0.77	392,310.55	0.77	392,311.32
Red gum	1996	0.00	0.00	409,835.97	0.00	409,835.97
Red gum	1997	0.00	0.00	291,068.76	0.00	291,068.76
Red gum	1998	0.00	0.00	602,672.15	0.00	602,672.15
Red gum	1999	0.00	0.58	434,674.34	0.58	434,674.93
Red gum	2000	0.00	0.00	398,582.08	0.00	398,582.08
Red gum	2001	95.16	0.01	217,938.57	95.17	218,033.74
Red gum	2002	0.00	0.00	148,693.45	0.00	148,693.45
Red gum	2003	0.00	0.00	244,985.89	0.00	244,985.89
Red gum	2004	0.00	0.00	201,598.47	0.00	201,598.47
Red gum	2005	0.00	0.00	200,938.25	0.00	200,938.25
Red gum	2006	67.04	0.00	174,534.03	67.04	174,601.08
Red gum	2007	144.50	0.00	286,213.34	144.50	286,357.85
Red gum	2008	0.00	3.25	177,813.26	3.25	177,816.52
Red gum	2009	0.00	1.01	345,544.98	1.01	345,546.00
Red gum	2010	0.00	0.00	719,668.16	0.00	719,668.16
Red gum	2011	0.00	52.89	625,584.78	52.89	625,637.67
Red gum	2012	149.40	1.72	399,836.92	151.12	399,988.04
Red gum	2013	0.00	0.00	283,978.08	0.00	283,978.08
Red gum	2014	679.19	0.36	205,471.56	679.55	206,151.12
Red gum	2015	0.00	18.08	201,740.37	18.08	201,758.45
Red gum	2016	0.00	0.00	468,592.30	0.00	468,592.30
Red gum	2017	0.00	0.01	222,438.15	0.01	222,438.16
Red gum	2018	0.00	0.23	179,895.41	0.23	179,895.63
Red gum	2019	0.00	2.37	249,457.76	2.37	249,460.13
Red gum	2020	165.33	0.00	307,612.48	165.33	307,777.80
Red gum	2021	0.00	0.00	518,225.42	0.00	518,225.42
Red gum	2022					1,206,120.80

Black box

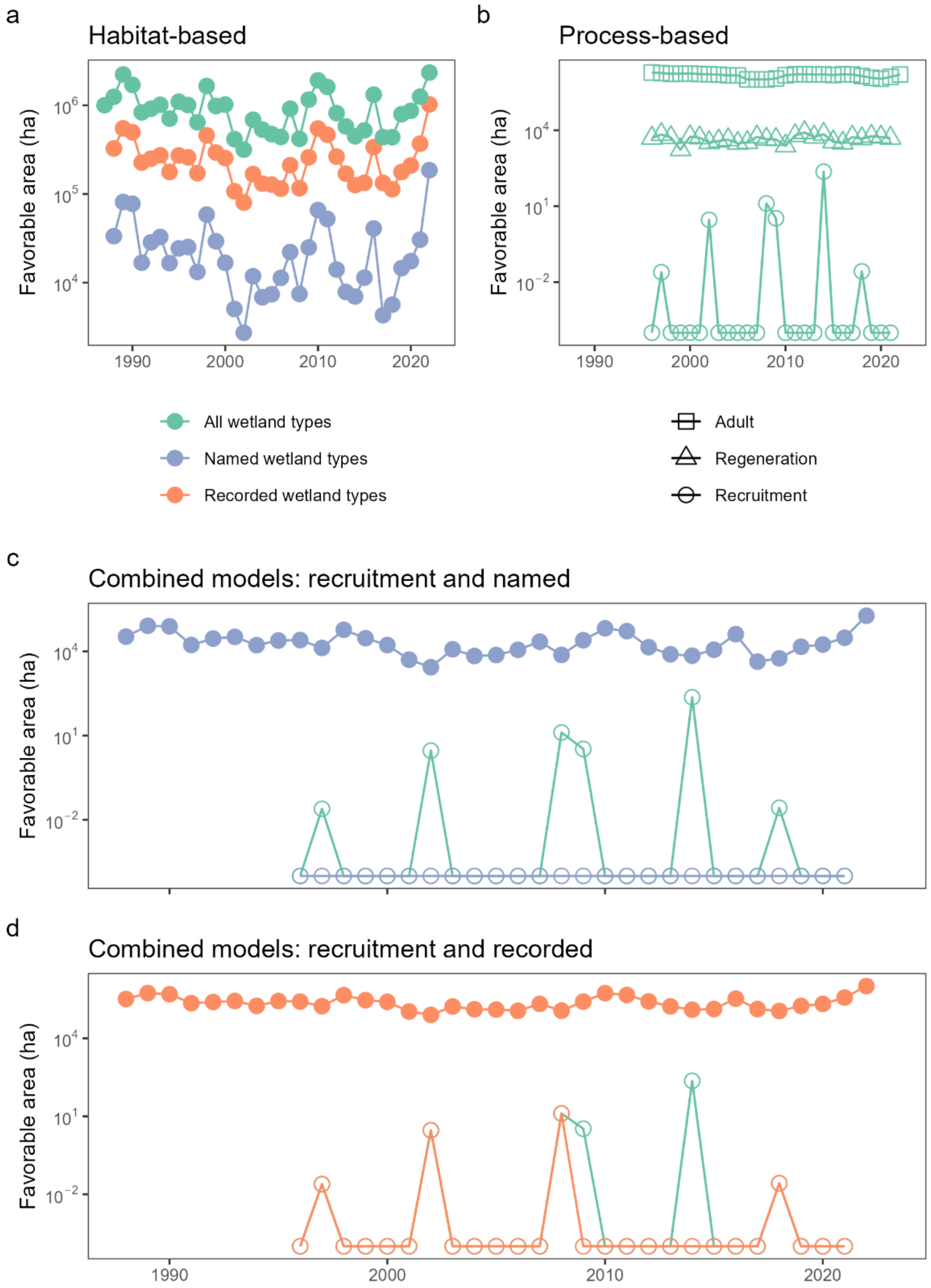

Figure S13. Temporal variation in the area identified as favorable for black box according to habitat-based, process-based, and combined models. For further details, see caption of Figure 6 in the text.

Coolabah

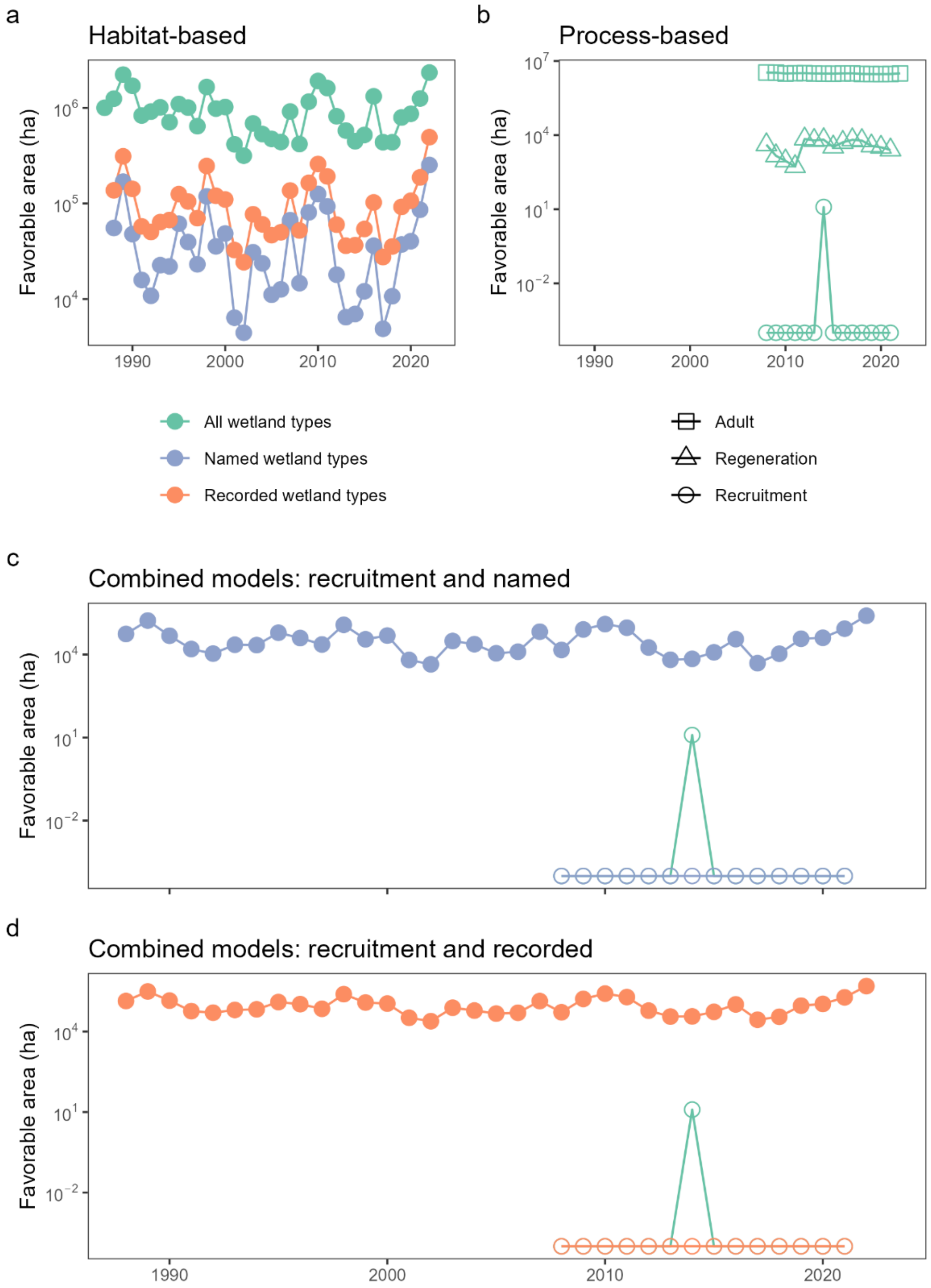

Figure S14. Temporal variation in the area identified as favorable for black box according to habitat-based, process-based, and combined models. For further details, see caption of Figure 6 in the text.

Lignum

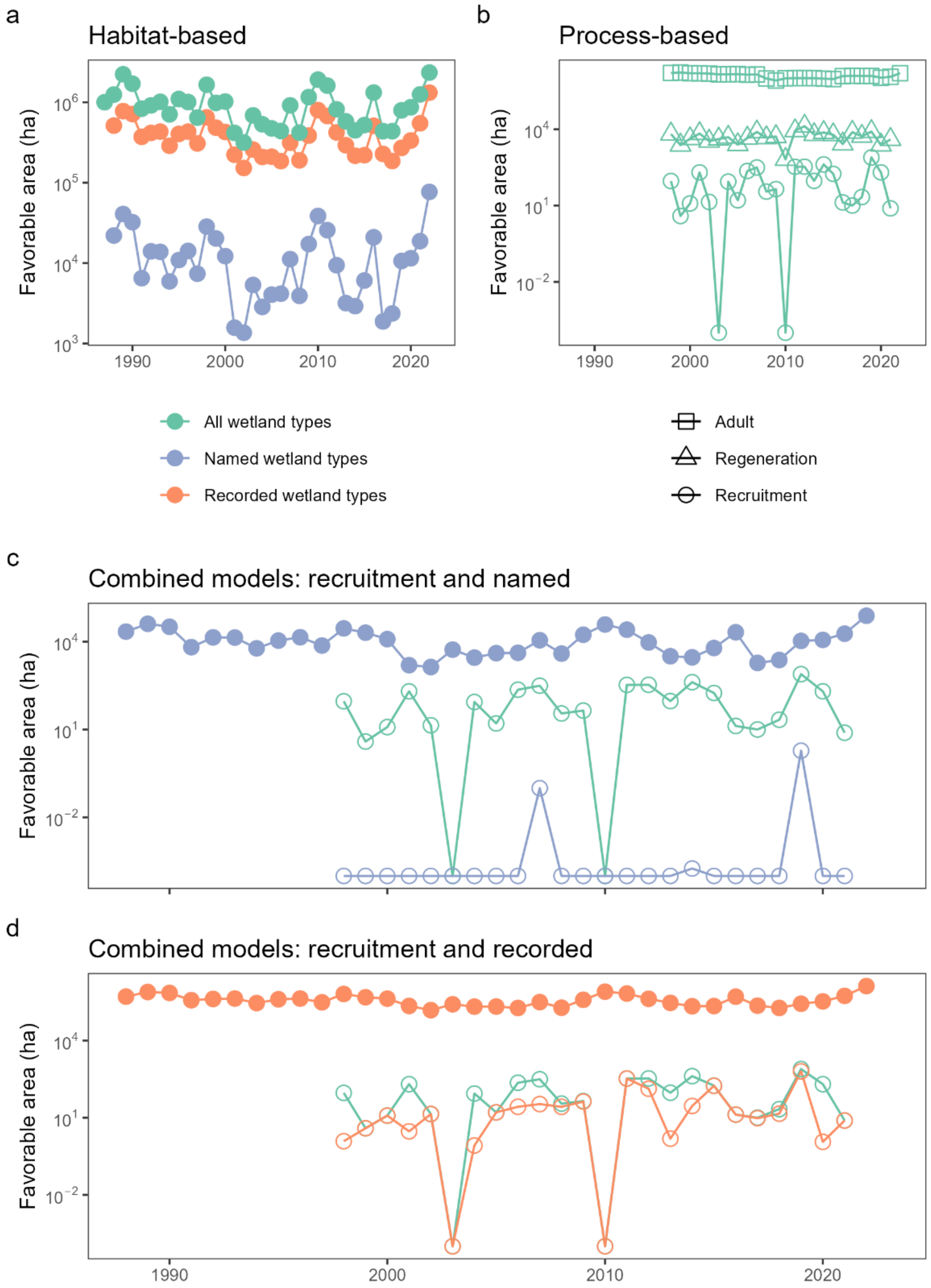

Figure S15. Temporal variation in the area identified as favorable for black box according to habitat-based, process-based, and combined models. For further details, see caption of Figure 6 in the text.
